# Reconstruction of 3D crystal growth from transmission optical microscopy images

**DOI:** 10.1093/pnasnexus/pgag080

**Published:** 2026-03-24

**Authors:** Thomas P Ilett, Thomas A Hazlehurst, Chen Jiang, Cai Y Ma, David C Hogg, Kevin J Roberts

**Affiliations:** School of Computer Science, University of Leeds, Leeds LS2 9JT, United Kingdom; School of Computer Science, University of Leeds, Leeds LS2 9JT, United Kingdom; School of Chemical and Process Engineering, University of Leeds, Leeds LS2 9JT, United Kingdom; School of Chemical and Process Engineering, University of Leeds, Leeds LS2 9JT, United Kingdom; School of Computer Science, University of Leeds, Leeds LS2 9JT, United Kingdom; School of Chemical and Process Engineering, University of Leeds, Leeds LS2 9JT, United Kingdom

**Keywords:** crystal morphology, crystallization processes, machine learning, computer vision, 3D shape reconstruction

## Abstract

Variations in the 3D shapes of crystalline materials can greatly affect their physical chemical properties, influencing the downstream processes required for their formulation, precision manufacturing, and transportation. Under different growth conditions, identical compounds can produce a wide variety of particle shapes and sizes, yet a comprehensive understanding of the relationship between crystallization conditions and final product properties is severely lacking. In part, this reflects the technical challenges associated with accurately recovering 3D crystal shape information during their growth. Here, we present a novel method for reconstructing the evolving 3D polyhedral shape of a single crystal in a growth cell from a sequence of transmission optical microscopy images. Our approach combines deep learning with synthetic image generation based on theoretically grounded crystallography together with accurate simulation of the refraction of light at the crystal faces, yielding robust estimates of the dynamics of the crystal shape evolution during the crystallization process. We demonstrate our approach by tracking the 3D growth and shape development of the polyhedral *α* form of L-glutamic acid and validate our results against manual measurements collected using a purpose-built interactive software tool. We observe changes in the relative areas of crystal faces, characterizing not just the size but also the shape changes during growth. This approach offers a new framework for in situ monitoring of crystal growth and may support future advances in the precision manufacturing of crystalline particles through the improved digital design and control of crystallization processes.

Significance statement Crystallization processes are ubiquitous in society, the environment, and in industry, where nearly 80% of chemicals involve processing crystalline solids. Reflecting their 3D structural anisotropy, crystals mostly take a polyhedral external form, which critically determines the material’s physical/chemical properties. Developing the necessary understanding and control of crystallization in real-time ensures product consistency. Hence, there is an essential need to track the 3D shape and size of crystals during growth in response to changing process variables such as growth solution temperature and supersaturation. To date, there has been no effective way of doing this. To help address this need, we present a novel machine learning method for tracking the 3D growth of *α*-LGA crystals from sequences of transmission optical microscopy images.

## Introduction

A substantial proportion of medical, industrial, and agricultural chemicals are produced, processed, stored, and transported in faceted crystalline form (1). The size and shape of the crystal particles is important from a practical point of view, for example, as to how well they can be manufactured, processed, packed, and stored. However, for many compounds, these properties also significantly impact the ultimate chemical reactivity of the particles and how these interact within their target processing and product end-use environments. At best, a proportion of the product may be less effective, at worst, harmful to humans or the environment. While particle size has historically been the principal focus for particle process optimization and control, there has recently been a growing awareness of the need to also monitor and hence control particle shape during the crystallization process. The shape of a crystal and its overall physical chemical properties depend on how its bulk crystallographic structure is exposed on the crystal’s habit surfaces and how they develop during the growth process (1). Extracting crystal shape information during crystallization is particularly challenging. The use of camera probes typically produces poorly defined images because the crystals are passing by the cameras quickly with various orientations and can often be fragmented or agglomerated. As a result, shapes are often crudely characterized with simplistic descriptors such as needle-like, plate-like, or spherical. While these can provide useful insights, such characterizations cannot help us to investigate and understand theoretically grounded questions about the crystal’s surface-specific interactions and their impact on particulate properties. In this work, we develop a tool that will help us do just that. By constructing a complete 3D polyhedral shape of a crystal, we know the surface areas of individual faces and, when the crystallographic structure of the compound is known, physical chemical properties such as the surface structure and energies can be directly calculated.

The 3D reconstruction of objects and scenes from images has been an important research topic for many years. Early work focused on the recovery of shape from two or more views, obtained from multiple cameras, or from a single moving camera (2). Shape can also be recovered from a single view by exploiting prior models of the allowable geometry, including limited variations in size and structure (3). More recently, machine learning has unified many sources of information on shape, known and unknown, by directly learning a mapping from an image to a depth map of the visible surfaces (4). A neural network can be trained on pairs of images and depth maps, using natural images and active sensors (5) to obtain the depth map, or synthetically generated pairs of images and depth maps (6).

In the crystal domain, the shape of a crystal with a known crystallographic structure is highly constrained. As a polyhedral shape with *m* faces, a crystal can be represented by the orthogonal distance from a crystal-centered origin to each face (*m* degrees of freedom, *dof*). To model the projection of a crystal onto an image plane, we also require the “pose” of the crystal with respect to the camera—its position (3 dof) and orientation (3 dof). The shape and pose together give m+6 degrees of freedom in total. Assuming all *n* vertices (front and back) are visible and ignoring refraction for the moment, we obtain two equations in the m+6 unknowns for each vertex from its projection into the image plane of the camera, which we assume to be calibrated. Thus, assuming 2n≥m+6, we can in principle recover the shape and pose of the crystal from the projected vertices. Given an initial estimate, the recovery can be achieved through nonlinear optimization of the m+6 pose and shape parameters to align the *n* crystal vertices with their projections in the image, through a form of “bundle adjustment” (2). The actual situation is made more complicated by refraction of back vertices and edges, which are displaced in the image plane by the bending and scattering of light rays at the front faces of the crystal. We also observe that back vertices and edges often appear in more than one front face due to refraction, providing multiple views of the structure that we can exploit (eg Figure S2). In principle, the refraction can be modeled in updated projection equations.

The geometric approach outlined above depends on knowing the correspondence between crystal vertices and their projection onto the image plane, which may be problematic to obtain, particularly as the appearance and disappearance of faces during growth means *m* and *n* are unknown a priori and subject to change. Back vertices may also be partially obfuscated by the rough surfaces of front faces. The geometric approach also fails to exploit other shape cues such as shading differences across edges and the appearance of junctions formed around the projections of vertices. Furthermore, the locations where back edges reach the edges of front faces produce characteristic image features that again provide a source of information on 3D shape. Our approach seeks to exploit these additional sources of shape and pose information by learning a direct mapping from images to crystal shape and position. We then iteratively refine this configuration by rendering and comparing crystal images and keypoint maps of projected vertices and edge junctions with the microscope images and estimated keypoint coordinates.

We claim that the recovered configurations from our approach are unique physical reconstructions, up to the finite set of rotational and reflective crystal symmetries. The iterative refinement of pose and shape parameters aligns vertices and edges of the crystal with their appearance in the microscope image and is therefore solving for the m+6 unknown shape and pose parameters by a similar argument to bundle adjustment on the vertices alone. This technical justification is validated in part by extensive experiments on synthetic data in which well-matched solutions always come from the expected symmetry set. Experiments using physical measurements of a real crystals are consistent with this, but only indicative at this stage. Occasionally our automated method may converge on an incorrect solution, but when it converges to a good match the solution is always from the same symmetry set.

In-process crystal growth imaging for a population of crystalline particles within their dynamic processing environment, where their spatial position and orientation continually vary, is particularly challenging, and even capturing high-quality single views of individual crystals can be difficult. As a step toward addressing this, we focus on the growth of single *α*-form L-glutamic acid (*α*-LGA) crystals inside temperature-controlled growth cells (7), capturing high resolution microscope images (8, 9) at regular time intervals. We use transmission-mode lighting to ensure the back faces are clearly visible through the crystal. While this set-up could be extended to capture multiple views simultaneously, we restrict ourselves to a single view for two key reasons. Firstly, we want to stay close to the more challenging, yet ultimately more important, in-process case. Secondly, using standard, inexpensive lab equipment ensures broad accessibility, allowing other researchers to easily use our software.

Our method to accurately fit 3D crystal shapes to single images combines complementary computer vision approaches designed to capitalize on the refractive properties of crystals. The first stage uses a neural network to directly predict the shape, pose, material properties, and lighting of the crystal, which we refer to as the *configuration* of the crystal. It is impractical to obtain sufficient data for training the neural network from manual estimation of the configuration from real crystal images. Instead, we build a synthetic dataset of randomly generated *α*-LGA crystals based on the law of rational indices, in particular the BFDH approximation (see eg (1, 10)) using known lattice information and some weak morphological constraints to ensure a degree of realism. We sample a wide range of configurations, and use a full ray-tracing rendering package (11) to generate synthetic images. We include simulated interference (bubbles, scratches, and background noise) to ensure that the synthetic images are at least as challenging as our real images. This dataset of synthetic images paired with known configurations allows us to train a neural network to directly, and quickly (after training) predict configurations from images. Application of the trained model to real *α*-LGA transmission microscope images shows good domain transference, but as differences between the real and synthetic domains persist and neural networks can be sensitive to image perturbations, the initial parameter prediction is not perfect. However, this provides a good starting point from which to iteratively refine the solution.

The next stage of our approach takes the initial crystal configuration prediction, and generates a realistic image using the same renderer used to generate the synthetic dataset. As real crystals have variability in the surface texture, visible “ghosts” of the initial crystal seed, and the images frequently contain bubbles and other transient noise, direct comparison between rendered and real images is not feasible. Modeling the complete noisy scene is theoretically possible, but in practice too computationally expensive. Instead, we “denoise” the real image to yield a simpler target that is compared, pixel-by-pixel, with the rendered image to produce an error term that, by inverting the rendering pipeline, is iteratively minimized by updating the shape, pose, material, and lighting parameters using gradient descent. This direct “inverse rendering” approach works well when the initial guess is close to the solution but often fails to converge or gets stuck in local minima otherwise.

To improve robustness and reliability, we introduce an additional geometric target into the inverse rendering pipeline by using a projection of the polyhedral crystal wireframe. Our projector accounts for refraction of back (only visible through the crystal) edges through front (directly visible) faces, identifying intersections between all refracted and nonrefracted edge segments as keypoints. A neural network, pretrained on the synthetic dataset, detects likely keypoints in the image, and the 2D geometric distances between corresponding detected and projected keypoints form the basis of an additional loss term. While this loss alone is not usually sufficient to achieve a perfect fit due to the limitations of the detector network, it simplifies gradient computation by circumventing the full inverse rendering step. As such, it plays an important role in bringing the initial solution close enough for the inverse renderer to converge effectively on the final fit.

To evaluate our method, we require accurate, manually verified 3D measurements. Although various automatic, semiautomatic and manual software packages and techniques (7–9, 12–16) have been published for generating 2D measurements of a crystal as it appears in an image, none have been capable of generating 3D measurements. To fill this gap, we integrate our wireframe projector into an interactive software tool that enables a user to overlay a projected (and refracted) crystal wireframe onto a crystal image. Users can manually adjust the shape, pose, and refractive index to align the projected edges and keypoints to those in the image (Figure S5). While time consuming, this allows us to create a ground-truth baseline for evaluation, and furthermore offers a stand-alone tool for manual 3D crystal sizing and fitting. Finally, we perform preliminary experiments to validate the approach against 3D measurements obtained using a laser confocal microscope (Section S3).

Our contributions are (i) a fully automated method and open source implementation for tracking the 3D growth of a single crystal from a sequence of transmission optical microscopy images, demonstrated here on *α*-LGA, (ii) a method for generating randomized synthetic crystal configurations and corresponding realistic microscopy images, (iii) an evaluation of the method on nine *α*-LGA crystal growth sequences across a range of supersaturations, and (iv) an interactive software tool to facilitate manual fitting of 3D crystal shapes to images.

## Results

### Synthetic dataset

To address the lack of ground truth measurements for real crystal images, we generated a synthetic dataset of 100,000 *α*-LGA crystals for training and evaluating our method (Figure 2, Dataset S1, Methods). This dataset comprises images created from randomized crystal configurations with various sources of visual interference such as bubbles and internal crystal inhomogeneities, providing realistic and challenging test cases with known parameter values. The synthetic dataset enables us to train neural networks to invert the imaging process by estimating crystal configurations and locating relevant image features—*keypoints*—directly from noisy images. By simulating a wide range of challenging imaging conditions, models trained solely on synthetic images generalize effectively to real *α*-LGA microscopy images captured in our experimental setup despite not being exposed to them during training. The components developed to generate realistic synthetic images, along with the ability of neural networks trained on synthetic data to transfer their learning to real data, are fundamental both in making an initial prediction and in the subsequent refinement stages.

### One-shot parameter prediction

**Figure 1:**
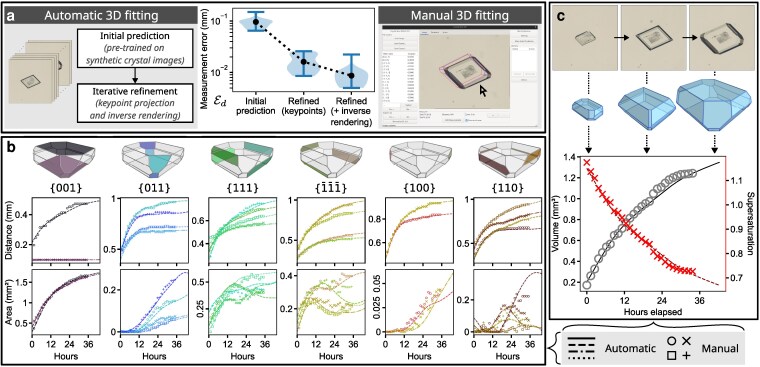
A sequence of 3D crystal shapes is automatically fitted to a series of 2D microscope images. a) The automatic fitting method is comprised of three components; an initial prediction followed by two refinement techniques. Each component significantly improves agreement with manual 3D measurements generated using our custom software tool. b) Automatic center-to-face distances and face areas (lines) show close agreement with manual measurements (symbols) across all crystal face groups. Nonmonotonic area development and the appearance/disappearance of faces suggests that concentration-dependent growth mechanisms vary between faces. c) Increase of crystal volume corresponds to a decrease of supersaturation during the growth process. Enlarged plots and screenshot of the software are included in Figure S7.

The first of the three neural network components is the predictor, trained to predict crystal configurations directly from noisy images. The predictor model is based on an established and state-of-the-art image classifier, RegNetZ (17), and trained on the synthetic dataset. RegNetZ offers a good balance between model size and generalization performance compared to other architectures. When compared with 28 manually measured crystal configurations from the growth sequence shown in Figure 1 (sequence 2 in Table S2), with mean measured center-to-plane distance 0.63 mm, the network achieves mean center-to-plane distance errors of 0.088 mm and mean vertex position errors of 0.192 mm (Methods, Table 1).

**Table 1:** One-shot prediction performance.

Model	# Params (M)	$E_{d}$ (mm)	$E_{v}$ (mm)
RegNetZ (17)	21.70	0.088	0.192
RegNetX (17)	6.63	0.166	0.330
RegNetY (17)	80.76	**0.066**	0.182
EfficientNet-B3 (27)	10.79	0.188	0.377
EfficientNet-B4 (27)	17.66	0.196	0.317
ECA-ResNet-D (28)	100.18	0.070	**0.180**
MobileNet-V4-Small (29)	2.58	0.308	0.479
MobileNet-V4-Large (29)	31.39	0.121	0.275
ViT-Small (30)	21.86	0.094	0.270
ViT-Base (30)	86.18	0.166	0.333
ViT-RelPos (31)	118.58	0.084	0.699

One-shot prediction performance relative to 28 manual measurements (best values in bold), errors reported in millimeters. Models were trained on the synthetic crystal images dataset for up to 100 epochs, with maximum wall-clock time of 48 h. Mean center-to-plane distance error: $E_{d}=\sum_{i}|d_{i}-{\hat{d}}_{i}|/|D|$, where ${\hat{d}}_{i}$ are the manual measurements. Mean 3D vertex position error: $E_{v}=mean(\min_{j}\Lambda_{ij},\min_{i}\Lambda_{ij})$, where $\Lambda_{ij}=\|v_{i}-{\hat{v}}_{j}{\|}_{2}$ is the pairwise matrix of Euclidean distances between predicted and target 3D vertices, with nearest-neighbor distances taken in both directions.

### Iterative shape refinement

Starting with a one-shot prediction of the crystal configuration, we quantify the goodness-of-fit to the target image by rendering an image of the predicted crystal and projecting the vertices. The rendered image is compared with a denoised version of the target image, and the projected vertices are compared with keypoints detected in the target image (Figure 3, Methods). The differences are combined and minimized using gradient descent optimization, automatically refining the 3D fit to the crystal image. The success of this stage depends on how close the initial estimate is to the solution, how much image information is lost through the denoising and how well the keypoints can be extracted from the target image. Low quality images, bubbles, or crystals with prominent defects can produce anomalous features that confuse the algorithm. In these cases, having a good initial estimate is particularly important, yet even in relatively noise-free scenarios, poor initialization can lead to slow convergence or entrapment in local optima.

Across unseen synthetic examples where 3D ground truth is known, refinement improves 3D vertex position accuracy by 90±10% (mean ± SD) after 300 iterations. Similarly, the error between known and predicted face distances improves by 94±12% (Figure S3). In most cases this yields almost-perfect matches. For the manual reference measurements from the growth sequence in Figure 1, we find that keypoint-only refinement improves measurement errors by 82±5% compared to the initial predictions. Including keypoints and inverse rendering during refinement reduces the errors further, by 91±3% (Figure 1a).

To characterize sensitivity to initialization, we repeat the refinement process above under identical optimization settings, initialized with controlled perturbations of the converged parameters (see Figure S4 for full details). We deem a run successful if, after 300 iterations, it returns to its reference solution within a threshold *τ* on the final mean nearest-neighbor vertex distance, normalized by crystal size. Return rates are generally robust to rigid-body misalignment: at τ=0.1, 97–98% of runs return under moderate perturbations (a 10° 3D rotation or a 10% relative in-plane shift). Stricter thresholds expose greater sensitivity to morphological perturbations: for σ=0.1 center-to-plane distance noise, the return rate is 52% at τ=0.05. This highlights the importance of a good initial prediction to single-frame convergence. However, in sequence fitting, the learned temporal model can mitigate occasional poorly initialized frames by favoring a smooth, globally consistent trajectory over time.

### Crystal growth tracking

To track the 3D growth of a crystal across a sequence of images, we train a sequence generator (SG, a feed-forward neural network) to model the time-dependent growth behavior of the crystal. The SG is trained by iterative refinement on random frames drawn from the crystal growth sequence, producing a smooth and coherent reconstruction of the crystal configuration over time.

We apply our method to nine real *α*-LGA crystals grown under typical conditions (Methods), recovering individual face areas, crystal volumes and, by calculation (Section S1, (18, 19)), the changes in solute concentrations and supersaturations (Figure 1c, Methods). Of the nine crystal growth sequences, the automatic method produces seven near-perfect fits, and two partial fits where some front edges are confused with back edges. Table S2 gives details of the performance for each sequence. For the two partially fit sequences, we can recover the correct solutions by combining the automatic method with soft constraints derived from manual fits of a small number of selected frames (Methods, Figure S8). While requiring additional manual effort, semiautomatic fitting still significantly increases reconstruction throughput over a completely manual approach.

Two sequences produce partial fits in which a subset of apparent front edges are incorrectly matched to refracted back edges, forcing other faces away from their correct positions. This occurs when the image evidence is consistent with more than one plausible front/back-edge assignment. Small errors in the initial predictions or in the refinement targets (denoised images, detected keypoints), which can be caused by bubbles or crystal defects, steer the local gradient-based optimization toward an incorrect solution. In practice, we recover the correct solution by adding one or more manually fitted frames as soft constraints, which guides the sequence generator into the correct attractor while retaining a predominantly automated workflow.

Face growth dynamics at the crystal-solution interfaces are primarily governed by surface chemistry, which varies between crystal facet forms due to differences in interactions with the solution environment. However, substantial variation can also occur within these crystallographically equivalent form groups due to lattice defects such as dislocations, and/or local inhomogeneities in mass transfer for the different crystal facets. For example, in our experiments with *α*-LGA, the top face (100) is fully exposed to the solvent and hence grows differently to the bottom face $\left(\bar{1}00\right)$, which is mostly in contact with the bottom surface of the growth cell. Traditionally, estimating concentration-dependent face growth dynamics requires multiple experiments at different solute concentrations, aggregating results from linear fits to early-stage face growth during which supersaturation remains close to known initial values (7). Here, with supersaturation tracked throughout the sequence (Methods), the full information can be used to develop a precise mechanistic growth model from fewer experiments.

## Discussion

Understanding both the size and shape of crystals during crystallization processes is fundamental to creating effective products efficiently, and with minimal environmental impact. Here, we present a complete toolkit for fitting and tracking 3D crystal shapes over time from readily available microscope images of single crystals under transmission lighting and in situ within their crystallization environment. We demonstrate the toolkit by tracking the 3D shape development of *α*-LGA crystals grown under these conditions. Our approach leverages state-of-the-art computer vision techniques for automated shape estimation while also offering an interactive software tool for manual fitting.

These methods are specifically tailored for single crystals and are not immediately transferable to larger-scale crystallization processes. In such systems, issues such as image resolution, crystal motion/rotation, agglomeration and breakage introduce significant additional challenges to the isolation, imaging and 3D reconstruction of individual crystals. One possible approach that could support application of the current methodology is the diversion of slurry through a controlled flow cell to allow imaging of immobilized crystals under controlled optical conditions; however, this would require further validation to assess feasibility. While direct implementation in fully in-process crystallization systems remains challenging, expanding our understanding of how individual crystals grow and develop over time advances theoretical insight into their chemical properties and potential applications, and provides a mechanistic foundation for population-level modeling and future in situ measurement strategies.

The partial-fit cases suggest several directions to improve robustness. First, refinement relies on local, gradient-based optimization; running the fitting procedure from multiple initializations (at the frame and/or sequence level) and selecting the best result could reduce occasional convergence to a locally consistent but incorrect fit. Second, while we already provide a reliable semiautomatic fallback via soft constraints from a small number of manually fitted frames, future work could focus on automatically detecting globally inconsistent sequence fits and then triggering targeted alternative initializations (eg exploring competing front/back-edge explanations) to minimize manual input. Third, incorporating additional visual cues may help disambiguate front versus refracted edges, such as polarization-resolved imaging or alternative illumination settings (20). Finally, reducing uncertainty in the intermediate targets (denoising and keypoint detection)—for example via more artifact-diverse training or confidence-weighting of detected keypoints—and extending the optical model beyond a single global refractive-index parameter to capture anisotropic/polarization-dependent behavior where needed could further reduce ambiguity in the evidence that drives optimization, while retaining the accessibility of a single-view setup.

Overall, the ability to track the 3D shape evolution of crystals during growth has the potential to open up new possibilities for improving crystallization processes. Here, we observe diverse, nonmonotonic development of face areas, suggesting that concentration-dependent growth mechanisms differ between faces. Insights such as these into crystal shape development can provide valuable input to morphological population balance models, complementing their treatment of growth rates, hydrodynamics, and scale-up effects in practical environments.

## Methods

### Polyhedral crystal shapes

Crystal shapes are defined by a set of intersecting planes with unit normal vectors $N=\{N_{i}\}$ and plane distances (origin-to-plane offsets) $D=\{d_{i}\}$, with the crystal origin $P\in R^{3}$ chosen anywhere inside the polyhedron. The plane normal vectors are precomputed for the crystal type *ζ*, using lattice unit cell proportions and symmetry group information that is read from the Crystal Structures Database (21) (CSD) (using identifier LGLUAC02 for the *α*-LGA crystals used in our experiments). The symmetry group defines face groups $G=\{G_{k}\}$ containing crystallographically equivalent faces, which theoretically have the same distances, but in reality there is always some variation between these ($d_{i}≃d_{j},\;\forall \;d_{i},d_{j}\in G_{k}$). Intersection points for all combinations of three linearly independent planes are computed to form a cloud of $n^{\prime }$ candidate vertices, $V^{\prime }\in R^{n^{\prime }\times 3}$, and filtered to the *n* polyhedral vertices $V\in R^{n\times 3}$ by selecting only points that lie on the inside (ie closer to the origin) of all planes; $v\in V\;⟺\;v\cdot N_{i}\leq d_{i},\;\forall \;i$.

Multiple crystal configurations can produce the same polyhedral shape due to: (i) zero-area faces caused by the corresponding face-planes lying anywhere beyond the polyhedral envelope (ie the faces have “grown out”), (ii) rotational symmetries of the crystal type, and (iii) freedom in origin placement. A canonical morphology is obtained by resolving these ambiguities in turn (i) The distances of grown-out planes are reduced to the threshold of appearance (ie the planes touch the surface of the polyhedron): $d_{i}\leftarrow \max_{v\in V}\{N_{i}\cdot v\}$. (ii) Rotational symmetries are resolved by considering all symmetry-equivalent rotations defined by the crystal’s point group, selecting the rotation with the smallest angle relative to the identity, and applying the corresponding permutation to the plane distances. (iii) The origin is canonicalized by choosing the point *P* that minimizes $\|N\cdot P-D{\|}^{2}$. The vertices for each face are used to create a triangular mesh (by including an additional vertex at the face center), and face areas computed as the sum of the areas of the component triangles.

The canonical parametrization of shape and pose is thus {ζ,D,P,R}, where the rotation $R\in R^{3}$ is given in the axis-angle rotation representation.

### Synthetic dataset

**Figure 2:**
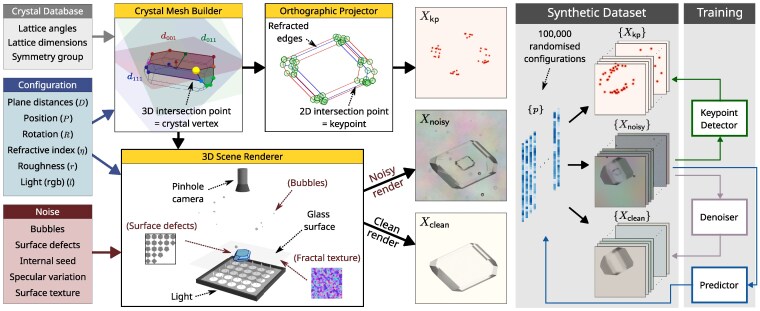
Components of the crystal projection and rendering pipeline used to generate the synthetic dataset. A 3D polyhedral mesh is constructed using face-plane angles and symmetries from the CSD combined with center-to-plane distances (*D*). The 3D shape is projected onto the image plane using an orthographic projector that accounts for refraction and yields geometrically significant keypoints—the intersection of any two (refracted or nonrefracted) line segments—that are then used to generate an image-like heatmap ($X_{kp})$. With the inclusion of additional material properties (roughness) and lighting, the 3D crystal is rendered into an image ($X_{clean}$) which can (optionally) be supplemented with the inclusion of a range of sources of randomized noise ($X_{noisy}$). A synthetic dataset is generated by randomizing parameters and noise, enabling the training of neural networks to map from $X_{noisy}\to X_{kp}$ (keypoint detector), $X_{noisy}\to X_{clean}$ (denoiser) and $X_{noisy}\to p$ (predictor).

We construct a digital twin of our laboratory microscope and growth cell setup using the differentiable rendering package Mitsuba (11), Figure 2. We define the geometry in a cell-centered coordinate frame with the *z*-axis pointing upwards toward the camera. From bottom to top this involves: an area light source pointing in the positive *z* direction (transmission-mode lighting), a glass surface on which the crystal sits, and a camera pointing in the negative *z* direction.

Crystal shapes are generated by drawing random distances from independent normal distributions for each face group ${\bar{d}}_{k}∼N(\mu_{k},\sigma_{k})$. To ensure randomized crystals retain a degree of realism, we include some weak constraints by defining a preferred ordering of distances and Zingg shape characterization bounds (22), and discarding random draws that violate any of these constraints. Asymmetry between the distances within each group are then introduced as $d_{i}∼N({\bar{d}}_{k},0.1{\bar{d}}_{k})$ for $d_{i}$ in group $G_{k}$, and then canonicalized to ensure nonappearing faces are at the threshold of appearance.

**Figure 3:**
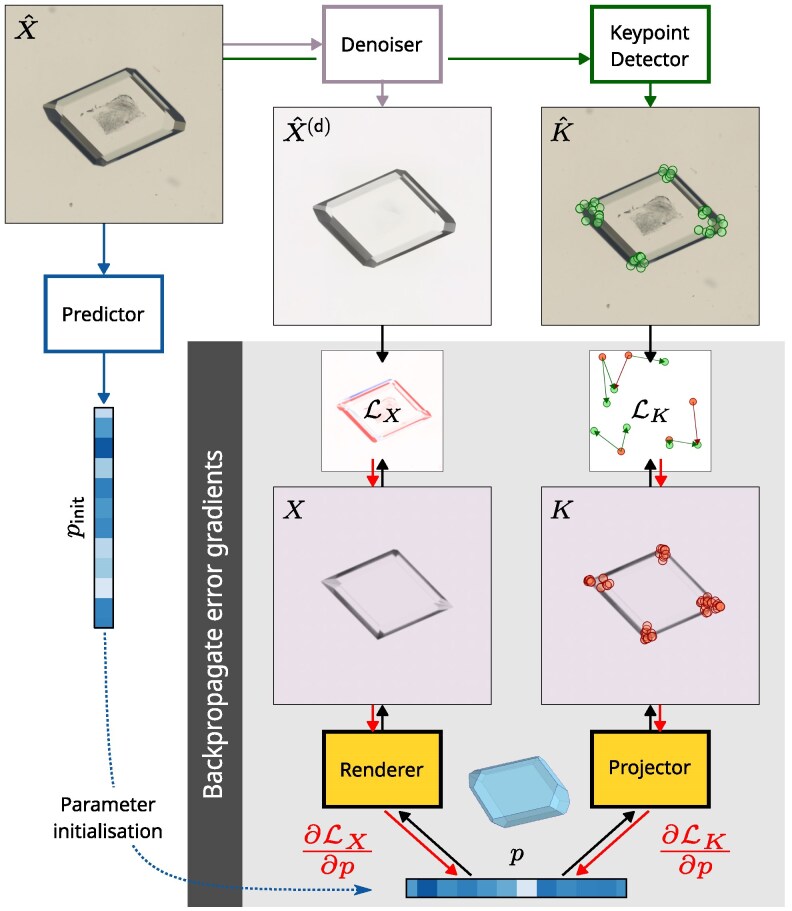
An initial estimate of the shape and scene parameters $p\leftarrow p_{init}$ is generated directly from the target image $\hat{X}$. The goodness-of-fit of the predicted parameters is determined by two main factors; how closely the rendered image *X* matches the denoised target image ${\hat{X}}^{\left(d\right)}$ and how close the keypoints of the projected predicted wireframe *K* are to the keypoints detected from the target image $\hat{K}$. Using inverse rendering and differentiable components, error gradients with respect to the parameters can be calculated using automatic differentiation.

Each crystal mesh is randomly rotated and translated, and then scaled so that the projected polyhedron is fully visible within the image with a projected area sampled from a uniform distribution, $a_{target}∼U(a_{min},a_{max})$. It must be fully appearing in the image and is constrained to always be touching the glass surface positioned at z=0 (Figure 2). The crystal material is modeled as a rough dielectric material with refractive index $\eta ∼U(\eta_{min},\eta_{max})$ and roughness $r∼U(r_{min},r_{max})$. The surface contains randomly generated line-defects using a bumpmap texture. Internally, a second crystal shape may be added with probability $\rho_{seed}$, representing the (typically visible, Figure 3) original crystal seed. The seed crystal shape is based on the main crystal with a reduced scale, plus small random perturbations to the distances and transformation. The surface of the seed is modeled with a smooth dielectric material with the same refractive index, *η*, and a randomly generated bumpmap noise texture to mimic the rough interface typically observed at the seed surface.

Further interference comes from bubbles (spherical meshes) that are added above and below the glass surface. Bubbles are randomly generated within set bounds for position, size and material properties, and cannot intersect other scene artifacts. The microscope is modeled as a perspective camera model with a thin lens and an aperture radius configured to keep the crystal in focus, while causing bubbles above and below the crystal to appear slightly out of focus, as seen in real images.

The light is parametrized by three (RGB) radiance values, $l\in [l_{\min},l_{\max}{]}^{3}$, on top of which sits a microfacet scattering layer with a specular transmittance texture that models the transition from the light source into the growth cell and introduces spatial variations in the color spectrum. The surface supporting the crystal is modeled as a glass sheet with additional normal map texture to mimic dirt, dust and other imperfections in and on the surface. A complete list of scene settings, constraints and bounds is included in Table S3.

Both noisy ($X_{noisy}$) and clean ($X_{clean}$) versions of the crystal scene are rendered at 400×400 pixel resolution. Additionally, for each random 3D crystal, we project the wireframe (with refraction) to the image plane to obtain 2D keypoints, *K*, and convert these into a corresponding heatmap image, $X_{kp}\in [0,1{]}^{400\times 400}$. The heatmap is composed of Gaussian blobs of height 1 and variance 5 px, centered at the keypoint locations and combined using the max operation, so that overlapping blobs do not exceed the maximum height of 1.

### Refracted wireframe projection

To find the predicted keypoint positions and to aid manual fitting of 3D shapes to real images, we project a polyhedral wireframe to the image plane using a projection that takes account of the refraction of back-facing edges through front-facing faces. Consideration of the refraction is essential for matching edges to the image as it is rarely obvious which of the observed edges are refractions and which are immediately visible.

Instead of mirroring the perspective camera with thin lens used for rendering, we use a simpler orthographic projection, scaled such that the projection of the glass surface onto the image plane is identical in both cases. The vertices are refracted through each of the visible faces according to Snell’s law, with each face acting as a window into the refracted positions of the hidden edges and vertices. Full details can be found in Section S2.

The final projected wireframe is comprised of many line segments connecting the refracted and nonrefracted vertices. The coordinates of the endpoints of each edge segment are collected to form the keypoints, *K*. By definition, the keypoints include the vertices, but they also include intersections between refracted back edges and the front edge of the face they are being refracted through. These locations are often easy to detect by eye, but their relationship to the refracted shape can be difficult to ascertain (Figure S2).

The physical accuracy of the 3D-to-2D mapping is supported by close agreement of projected edge-to-edge distances from fitted crystals with independent measurements made directly in the microscope software. Confocal measurements of out-of-plane heights show a larger discrepancy with our fitted mesh; however, axial distances in confocal microscopy can be sensitive to refractive-index mismatch and interface-related aberrations that distort the z-scale. We therefore interpret the confocal height comparison as an independent but indicative check rather than a definitive ground truth (Section S3, Figure S6, Table S1).

### One-shot predictor training

The predictor network is a regression from an input image to a crystal configuration. We trained and compared multiple neural networks across a range of different architectures and model sizes that are among the state-of-the-art for image classification on ImageNet (23), Table 1. Each model is initialized with pretrained weights from the huggingface repository (24, 25), and adapted for the task by replacing the final classifier layer with a fully connected layer that, after clipping to reasonable bounds, maps an image to a crystal configuration *p*, where |p|=|D|+3(position)+3(rotation)+2(material)+3(light), from which we calculate losses and optimize the network end-to-end.

The network is trained to minimize a composite loss between the predicted and target (canonicalized) crystal configurations. The composite loss is made up of plane distances $L_{d}=\|d^{pred}-d^{target}{\|}_{2}^{2}$, origin position $L_{P}=\|P^{pred}-P^{target}{\|}_{2}^{2}$, rotation (geodesic angle): $L_{R}=\arccos\;((\mathrm{tr}(R^{pred}R^{target⊤})-1)/2)$, material properties $L_{m}=(\eta^{pred}-\eta^{target}{)}^{2}+(r^{pred}-r^{target}{)}^{2}$, and light radiance $L_{l}=\|l^{pred}-l^{target}{\|}_{2}^{2}$. The combined loss is then $L=\omega_{d}L_{d}+\omega_{P}L_{P}+\omega_{R}L_{R}+\omega_{m}L_{m}+\omega_{l}L_{l}$, with weights $\omega_{\cdot }$ given in Table S4.

During training, synthetic images are augmented with random Gaussian blur and/or Gaussian noise. Models are trained for at most 100 epochs, or 48 h, whichever comes first, on a single V100 NVIDIA GPU using the AdamW (26) optimizer and other training parameters listed in Table S4. We use the rendering pipeline to visualize predicted configurations and for qualitative comparisons with ground truth images.

Although the models perform similarly on synthetic test data (Table S5), accuracy on our real *α*-LGA images varies widely (Table 1), suggesting that certain architectures and model sizes generalize to this real-image setup better than others. Based on these, we select the RegNetZ (17) convolutional neural network model for its model size and generalization performance. Inference is fast (predictor-only ∼0.017 s per image, including denoising and keypoint detection ∼3.0 s per image on a laptop NVIDIA RTX 4090 GPU; Table S7) and typically sufficiently accurate to ensure that subsequent iterative shape refinement converges to a good solution (Figure 1).

### Denoiser

The generation of corresponding pairs of clean and noisy images in the synthetic dataset provides us with two image domains that we can train a neural network to map between: $X_{noisy}\to X_{clean}$. Our denoiser is based on the MAGVITv2 (32) model that was developed to provide efficient mapping of images into a discretized token space suitable for use with large language models (LLMs), specifically for text-to-image synthesis. It consists of an image encoder that maps images into the token space and a decoder to generate images from the token space. We use an open-sourced implementation (Open-MAGVIT2, (33)) that has been pretrained on ImageNet (23), and then further train this model using an $L_{1}$ image loss: $L_{dn}=\text{mean}(|X^{\left(dn\right)}-X_{clean}|)$, where $X^{\left(dn\right)}=\text{denoise}(X_{noisy})$ and the mean is taken over the RGB image dimensions (400×400×3). We train the model for 10 epochs with the AdamW optimizer (26), using batch size 8 and learning rate $1e^{-5}$ (∼90 h on a single V100 NVIDIA GPU). See Table S4 for complete training parameters.

Real images are captured at 2,880×2,160 pixel resolution. To process these effectively and efficiently during inference on laptop hardware, the real image is cropped to square (2,160×2,160) and split into a grid of nine slightly overlapping subimages that are resized to 512×512, denoised separately, recombined with a linear blending of the overlapping regions and resized back to 2,160×2,160 to form the final denoised image. We found that the alternative of simply reducing the image resolution prior to denoising resulted in the loss of faint edges and poor results for smaller crystals.

### Keypoint detector

Similarly to the denoiser, the keypoint detector maps between image domains, in this case $X_{noisy}\to X_{kp}$, and is trained on synthetic data. We use the same base model (Open-MAGVIT2 (33)) and training regime as the denoiser (Table S4), but replace the $L_{1}$ image loss with an adaptation of the focal loss (34) that treats each output pixel as a probability of containing a keypoint. First, the binary cross-entropy loss is calculated pixel-wise: $L_{BCE}=-(X_{kp}\cdot \log\;(X_{kp}^{pred})+(1-X_{kp})\cdot \log\;(1-X_{kp}^{pred}))$ where $X_{kp}^{pred}=\text{predict}(X_{noisy})$, and the pixel values in $X_{kp}$ are treated as soft labels. The focal loss is then computed as: $L_{kp}=\text{mean}(\alpha \cdot (1-e^{-L_{BCE}}{)}^{\gamma }\cdot L_{BCE})$, where $e^{-L_{BCE}}$ represents the probability of predicting the correct class at each pixel, *α* is a weighting parameter for sparse positive examples, and *γ* is a modulating factor that adjusts the focus on hard-to-classify examples. The mean is taken over the image dimensions. We fix α=0.5 and γ=2 to account for the sparse distribution of small keypoint blobs in the heatmap-like images, ensuring that the loss focuses on these rare positive regions while down-weighting the contributions from easily classified negatives. We found that focal loss based on BCE loss with soft labels gave qualitatively better results and trained faster than using L1 or L2 losses.

Extraction of 2D keypoint coordinates ($\hat{K}$) from real crystal images $\hat{X}$ is done in two stages. In the first stage, we downsample the high resolution image to 512×512 and generate an initial keypoint heatmap ${\tilde{X}}_{kp}$. ${\tilde{X}}_{kp}$ is used to identify locations from which to select nine patches of size 700×700 to crop from $\hat{X}$ by iteratively convolving an averaging kernel of size 165×165 (where 165=700×512/2,160 is the downscaled patch size) over ${\tilde{X}}_{kp}$, selecting the location that yields the largest result and halving the values of ${\tilde{X}}_{kp}$ at that kernel location in that patch. Subsequent convolution iterations yield different patch locations that may overlap, but focus on the areas most likely to contain the keypoints. Next, the 700×700 cropped patches are downsampled to 512×512 and input to the keypoint detector to generate a batch of keypoint heatmap patches. The coordinates of the peaks within each heatmap patch are extracted,^a^ combined across patches, and nearby coordinates (within 20 px at full resolution) merged to form the final set of keypoints, $\hat{K}$.

### 3D shape refinement

Given a real crystal image $\hat{X}$, a denoised copy, ${\hat{X}}^{\left(dn\right)}=denoise(\hat{X})$, extracted keypoints $\hat{K}=detect\_keypoints(\hat{X})$ and an initial parameter prediction from the one-shot predictor $p_{init}=predict(\hat{X})$ (Figure 3), the goal is to improve the crystal configuration so it more accurately corresponds to the crystal in the image. We initialize the parameters $p\leftarrow p_{init}$, build the corresponding 3D polyhedron, identify keypoints (*K*) in the image plane, and render an image, *X*, using the differentiable renderer (Mitsuba (11)). The rendered image is compared against the denoised image to produce a pixel-wise $L_{2}$ image loss: $L_{X}=mean(|X-{\hat{X}}^{\left(dn\right)}{|}^{2})$. The predicted and target keypoints are compared to produce a second loss: $L_{K}=sinkhorn(K,\hat{K})$, where sinkhorn is a geometric optimal transport loss calculation (35). We also include additional regularization terms: $L_{z}=(\min\{v_{z}{\}}_{v\in V}{)}^{2}$ for ensuring the smallest *z*-component of the crystal vertices touches the surface at z=0; $L_{R}=R_{x}^{2}+R_{y}^{2}$, where $R_{x}$ and $R_{y}$ are axis-angle rotation components, to minimize crystal rotation out of the *xy*-plane; and $L_{o}={mean}_{i}(\max(0,d_{i}-{\bar{d}}_{i}{)}^{2})$, where ${\bar{d}}_{i}=\max(N_{i}\cdot V^{T})$, to ensure faces with zero area (ie grown-out faces) are on the threshold of appearance. The losses are combined in a weighted sum, $L_{ref}=\omega_{X}L_{X}+\omega_{K}L_{K}+\omega_{z}L_{z}+\omega_{R}L_{R}+\omega_{o}L_{o}$, to compensate for the differing orders of magnitude of the various loss terms (see Table S6 for values). The gradient of the loss with respect to the parameters, $\partial L_{ref}/\partial p$ is calculated automatically using PyTorch (36) and can be used to iteratively update *p* using a gradient descent optimizer (we use Adam (37)).

### Sequence fitting

**Figure 4:**
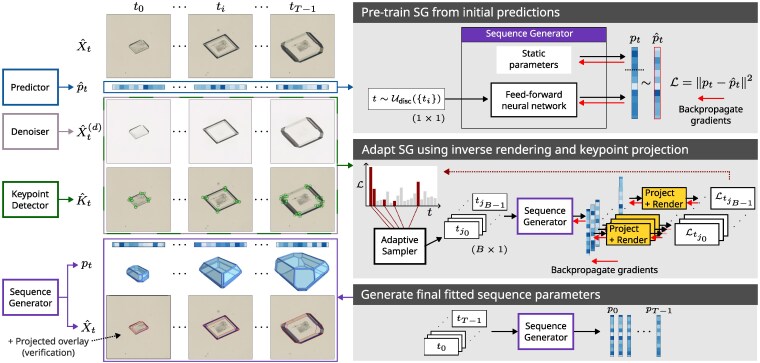
A sequence of images capturing the growth of a crystal is used to train an encoding of the sequence. First, the Predictor network generates initial parameter estimates for the entire sequence that are used to pretrain the SG. Next, the SG is trained to improve the predicted parameters by sampling batches of frames from the sequence, generating a corresponding batch of parameters, then projecting and rendering each one and using the error gradients (see Figure 3) to update the SG. An adaptive sampler is used to stochastically select frames with higher errors. After training, the final fitted sequence parameters are recovered from the SG by inputting the complete series of time points.

We extend single crystal fitting to a sequence of *T* crystal images ${\hat{X}}_{t},t=0,\ldots ,T-1$ by training a SG to learn the temporal dynamics of the parameters; $p_{t}=SG(t)$, Figure 4. The parameters are divided into two types: static (ie constant throughout the sequence) and dynamic. The rotation *R*, material roughness *r* and light radiance *l* are static, and the distances *D* are dynamic. For comparability with the manual measurements, we fix the origin position *P* and refractive index (*η*) to the manual values. The sequence generator normalizes the input time point $t^{\prime }=t/(T-1)\in [0,1]$ and passes it through a four-layer feed-forward network, employing GELU (38) activation functions, followed by a linear projection to the output vector of dynamic parameters (the distances, *D*). All refinement training parameters are listed in Table S6.

To train the SG, we generate initial predictions ${\hat{\;p}}_{t}$, denoised images ${\hat{X}}_{t}^{\left(d\right)}$ and target keypoints ${\hat{K}}_{t}$ for all *t*. We pretrain the SG to encode ${\hat{\;p}}_{t}$ using the loss function: $L_{pre}=\|p_{t}-{\hat{\;p}}_{t}{\|}^{2}$ since this does not require any crystal building or rendering and is thus very fast. We train for 1,000 batches of 256 time points randomly drawn from a discretized uniform distribution $U_{disc}(0,T-1)$ using the AdamW optimizer (26) and learning rate $1e^{-3}$. After pretraining, we sample batches (size *B*) of time points adaptively, by considering the errors across the sequence as a multinomial probability distribution and sampling without replacement in order to focus training on frames with higher errors. The errors for each frame *t* with parameters $p_{t}$ in the batch are calculated using a single step of the shape refinement process described above (Figure 3). One additional regularization term is included to penalize predictions of negative distance growth across the batch: $L_{+}=mean(\{\min(0,d_{i,t_{g}}-d_{i,t_{h}}{)}^{2},\;\forall \;t_{g},t_{h}\in B\text{ with }t_{g}>t_{h}\})$. The batch loss is thus $L_{B}=mean\{L_{ref,t}{\}}_{t\in B}+w_{+}L_{+}$ where $w_{+}$ is a constant weighting term, and the complete sequence error is given as: $L_{seq}=mean\{L_{ref,t}{\}}_{t=0,\ldots ,T-1}$. The SG is trained end-to-end by backpropagation of the errors through the projection and rendering stages and onto the feed-forward network parameters. The learning rate is initialized at $1e^{-4}$ and decreased by a factor of 0.8 when $L_{seq}$ has not improved for 200 steps. As inverse rendering is by far the most computationally demanding component, we first train for 1,000 steps using only the keypoint targets (plus regularization terms), then for a further 2,000 steps with inverse rendering included. Timings and GPU memory requirements for the main pipeline components (one-shot, refinement, and sequence fitting) are summarized in Table S7.

In the cases where fully automatic fitting results in incorrect edge matches (eg Figure S8), we are able to recover the correct sequence match by including one or more manually fitted crystal shapes as soft constraints on the fit. We achieve this by supplementing the refinement loss $L_{ref,t}$ with an additional term, $L_{man,t}={mean}_{i}\{(d_{i,t}-d_{i,t}^{man}{)}^{2}\}$, where $d_{i}^{man}$ is the manual measurement for face-distance *i* at frame *t*, included only for frames where manual measurements are available. This yields a robust, semiautomatic fallback option that ensures accurate sequence fitting can still be recovered in challenging edge cases.

### Interactive tool and user interface

Our interactive software tool integrates the wireframe projector with controls to manually adjust shape, pose, and refractive index properties. First, a crystal image and a starting crystal configuration are loaded into the tool. The 3D wireframe is projected on to the image using the method described above, taking refraction into account, so the shape can be compared directly to the image. The configuration can be adjusted interactively to update the projected wireframe in order to find a better fit to the image (see Figure S5). We found that the best fits for our *α*-LGA crystal images were obtained using a refractive index of 1.7, which is slightly larger than reported values (around 1.5–1.6, (39)). However, these values were obtained using *β*-form crystals suspended in oils, under very different optical conditions to ours.

Manual measurements from this interactive tool can be used as soft constraints for the automatic fitting (see Sequence fitting above). This hybrid approach provides a “human-in-the-loop” semiautomatic approach requiring minimal human assistance to correctly fit challenging crystal growth sequences (Figure S8, Table S2).

### Experimental apparatus and protocols

A crystal growth cell with temperature control provided by a recirculating thermostatic bath was set up with a Keyence VHX7000 digital microscope using transmission-mode lighting to capture high-quality images of a single *α*-LGA crystal (for further experimental details, see (7, 19)). An LGA solution saturated at 43 °C was prepared and transferred to a cuvette containing a single preprepared *α*-LGA crystal as the initial seed. The growth cell containing the cuvette was then cooled to a constant target temperature (Table S2). Crystal images were captured at 5 min intervals. Eight sequences contained 200 images (≃17 h) and one longer sequence contained 522 images (≃44 h).

## Supplementary Material

pgag080_Supplementary_Data

## Data Availability

The complete open source implementation of the method and associated tools (*CrystalSizer3d*) is available at: https://github.com/Shape4PPD/crystalsizer3d. The dataset of synthetic *α*-LGA crystal configurations and images used in this article is available at: https://doi.org/10.5518/1684.

## Notes

a Peaks are extracted using the peak_local_max function from the scikit-image python library (40).
