# Supplementary material for: Reconstruction of 3D crystal growth from transmission optical microscopy images
Source: PNAS Nexus. 2026 Mar 24;5(4):pgag080. doi: 10.1093/pnasnexus/pgag080 (PMC13070466; doi:10.1093/pnasnexus/pgag080)
Supplement: pgag080_Supplementary_Data [file pgag080_supplementary_data.zip › PNASNEXUS-PNASNEXUS-2025-01512R-s06.pdf]

Supporting Information for

Reconstruction of 3D crystal growth from transmission optical microscopy images

Thomas P. Ilett, Thomas A. Hazlehurst, Chen Jiang, Cai Y. Ma, David C. Hogg, Kevin J. Roberts

Kevin J. Roberts
E-mail: k.j.roberts@leeds.ac.uk

This PDF file includes:

Supporting text
Figs. S1 to S8
Tables S1 to S7
Legends for Movies S1 to S5
Legend for Dataset S1
SI References

Other supporting materials for this manuscript include the following:

Movies S1 to S5
Dataset S1

Supporting Information Text

S1. Calculation of concentration and supersaturation

As there is only a single crystal growing in the cuvette at any one time, the solute concentration at time t (C_t) can be calculated from the initial concentration ($C_0 = 16\,\mathrm{mg\,mL^{-1}}$) and the increase in the crystal volume (Φ) due to absorption of the solute (9):

$$C_t = C_{t-1} - \frac{(\Phi_t - \Phi_{t-1})\,\rho}{\Theta},$$

where $\Theta = 0.5\,\mathrm{mm^3}$ is the volume of the cuvette and $\rho = 1.54\,\mathrm{g\,cm^{-3}}$ is the crystal density (12). Crystal volumes are calculated from the polyhedral shapes using the trimesh python library (2).

The relative supersaturation at time t, denoted σ_t, is defined as the ratio of the concentration difference $C_t - \kappa$ to the solubility κ (with $\kappa = 7.36\,\mathrm{mg\,mL^{-1}}$ at the given temperature (6)):

$$\sigma_t = \frac{C_t - \kappa}{\kappa}.$$

S2. Calculation of refraction

We assume orthographic projection and a camera coordinate system with $x - y$ axes in the image plane and z axis pointing downward towards the crystal (see Fig. S1). We compute the refraction for a 3D back vertex v as it appears in a given front face of the crystal. The plane containing this face is defined by its unit normal N, pointing out of the crystal, and perpendicular distance to a given origin.

We seek the 3D displacement δ_v of the apparent position of the vertex without refraction v' to its actual position v:

$$\delta_v = v - v'.$$

Since the apparent position could be at any depth, we choose the point at the same depth from the image plane as v so that the displacement is a vector parallel to the image plane (see Fig. S1). As both T and N are known, we have $\cos\theta_t = T \cdot N$. From the known refractive indices of the crystal η_i and surrounding suspension η_t, Snell's law (1) states that:

$$\eta_i \sin(\theta_i) = \eta_t \sin(\theta_t),$$

where θ_i and θ_t are the angles between the unit normal to the plane N, the unit directions I and T of the incident ray from the vertex v and the transmitted ray towards the camera, respectively. From Snell's law it follows that light is only refracted up to a critical angle of incidence such that $\sin\theta_i \leq \eta_t/\eta_i$; beyond this, an incident ray is totally reflected back into the crystal.

Due to our assumption of orthographic projection, $T = (0, 0, -1)$. Following the construction in (3), the magnitude of the components of T and I tangential to the face plane are given by:

$$\|T_\parallel\| = \sin\theta_t, \text{ and}$$
$$\|I_\parallel\| = \sin\theta_i.$$

Thus, from Snell's law:

$$\|I_\parallel\| = \eta\|T_\parallel\|,$$

where $\eta = \eta_t/\eta_i$ is the ratio of the refractive indices of the suspending medium and the crystal.

Because $T_\parallel$ and $I_\parallel$ point in the same direction, we get:

$$I_\parallel = \eta T_\parallel = \eta\,(T - (T \cdot N)N) = \eta(T - \cos\theta_t N)\,.$$

The component of I orthogonal to the face plane is simply $I_\perp = \cos\theta_i N$. Thus, the incident ray I is given by:

$$I = I_\parallel + I_\perp = \eta(T - \cos\theta_t N) + \cos\theta_i N = \eta T + (\cos\theta_i - \eta\cos\theta_t)N$$

and

$$\cos\theta_i = \sqrt{1 - \sin^2\theta_i} = \sqrt{1 - \eta^2\sin^2\theta_t} = \sqrt{1 - \eta^2(1 - \cos^2\theta_t)}.$$

Thus, the distance h_N from v in the direction of I to the face plane is given by:

$$h_I = \frac{h_N}{\cos\theta_i},$$

where h_N is the orthogonal distance from the vertex to the face plane.

Finally, the displacement δ_v is given by:

$$\delta_v = h_I\,(I - (I \cdot T)T)\,.$$

Note that the incident direction I is the same for all vertices, since we assume an orthographic projection. Only the computed distance h_I varies between vertices, which, in turn, determines the magnitude of the displacement.

For each front crystal face, the vertices located behind it are visible by refraction. Edges connecting these vertices are drawn, but any portion extending beyond the face's boundaries is truncated. Intersection points with the edges of the refracting face become new end points for truncated edges. Vertices that do not appear within the face are discarded.

S3. Validation study using Laser Confocal Microscopy

We performed a preliminary comparison of measurements obtained from our manual tool with measurements from a laser confocal microscope (Fig. S6). Measurements were obtained from a single α-LGA crystal in air, and from the same crystal flipped over. We found a refractive index of 1.5 was needed to obtain a good manual fit for this simpler setup. In-plane measurement values were obtained from 2D edge-to-edge distances measured using the Keyence VHX7000 microscope's built-in line tools and pixel-to-length calibration (no feature detection or model fitting); see panel (a) in Fig. S6. Axial measurements were taken from a Keyence VKX3000 laser confocal microscope for the same crystal (panel (b) in Fig. S6).

The results in Table S1 show close agreement for 2D in-plane measurements. The level of agreement on axial height is more varied: three faces ($(111), (1\bar{1}\bar{1})$ and $(0\bar{1}1)$) are within 5% of the confocal measurements, three faces ($(\bar{1}\bar{1}1), (\bar{1}1\bar{1}), (011)$) plus the overall height are within 20.2%, and the remaining faces ($(\bar{1}\bar{1}\bar{1}), (0\bar{1}\bar{1}), (01\bar{1})$ and $(11\bar{1})$) show differences between -34.5% and $+61.9\%$. Multiple confocal measurements were obtained for two faces for which we observed significant variations in the confocal measurement values: $(0\bar{1}\bar{1})$; 97 µm and 152 µm, and $(01\bar{1})$; 141 µm and 198 µm. These variations amplify the differences with our manual measurements (we report worst-case), but we note that in both cases our manual measurements lie within the confocal ranges.

In laser confocal height scanning, surface topography is measured by detecting the intensity of light reflected from the focal plane on the object. The position corresponding to the maximum reflected light intensity is interpreted as the surface height. Therefore, the measurement accuracy relies on the intensity of the reflected light. For transparent or semi-transparent materials especially, this may lead to a lot of noise in the scanning results. In practice, we observed the laser confocal scan struggled to obtain accurate measurements for small-area faces in the crystal, because the tilted edges of those faces are close together and difficult to separate under the interference of noise. As a result, different scans on the same face may be quite different. On the other hand, the results on several big faces provided convincingly clear and accurate measurements. Thus, we interpret the confocal comparison as an indicative check rather than a definitive ground truth.

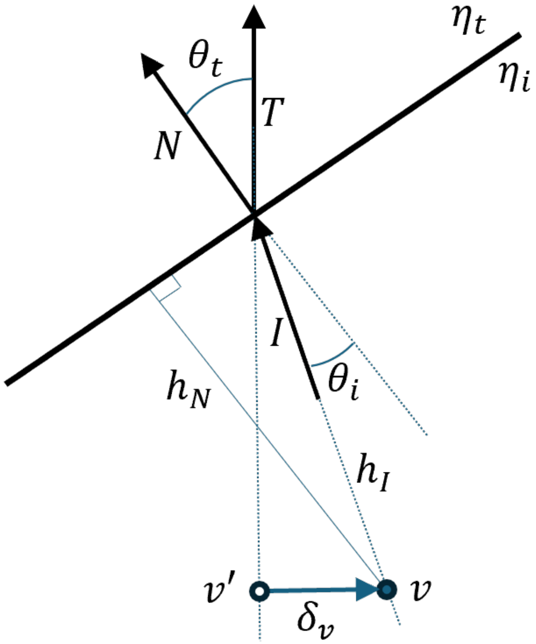

Fig. S1. A vertex v is refracted when viewed through a crystal face. The unit vectors of the incident and transmitted rays are denoted I and T respectively. These unit vectors make an angle of θ_i and θ_t with the unit normal to the face N. Assuming orthographic projection in the direction T, we aim to trace backwards from the known direction T to the unknown direction I using Snell's law. Vertex v is perceived to be at $v' = v - \delta v$. The refractive indices of the crystal and surrounding suspension are η_i and η_t respectively.

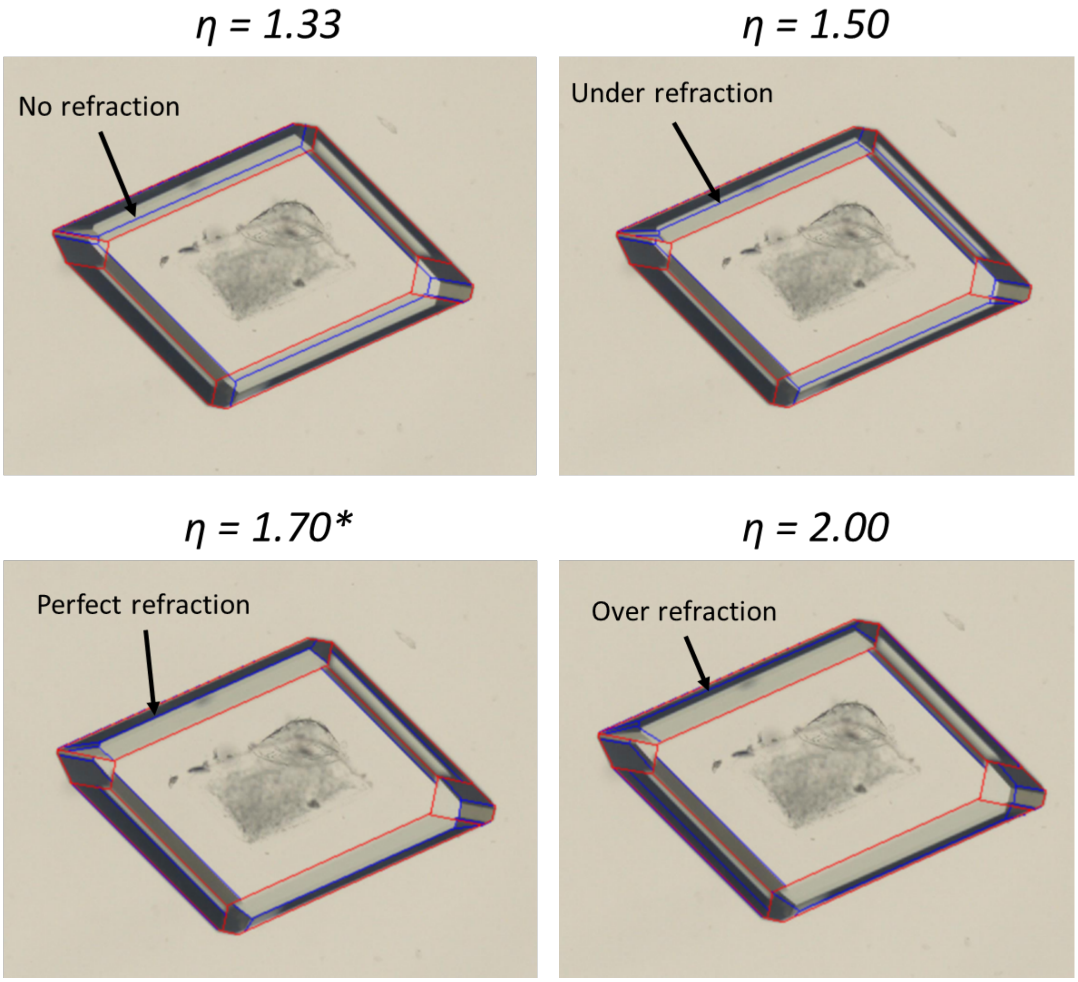

Fig. S2. A comparison of a real crystal image overlaid with the projection of the same 3D shape, where only the index of refraction (η) is changing. At $\eta = 1.333$ (equal to the refractive index of water, which we set as the external medium), no refraction occurs, $\eta = 1.5$, some refraction occurs, but not enough, $\eta = 1.7$ a perfect fit (indicated with $*$), $\eta = 2$ too much refraction occurs. Note that the red lines are directly visible and hence are not affected by the change in η.

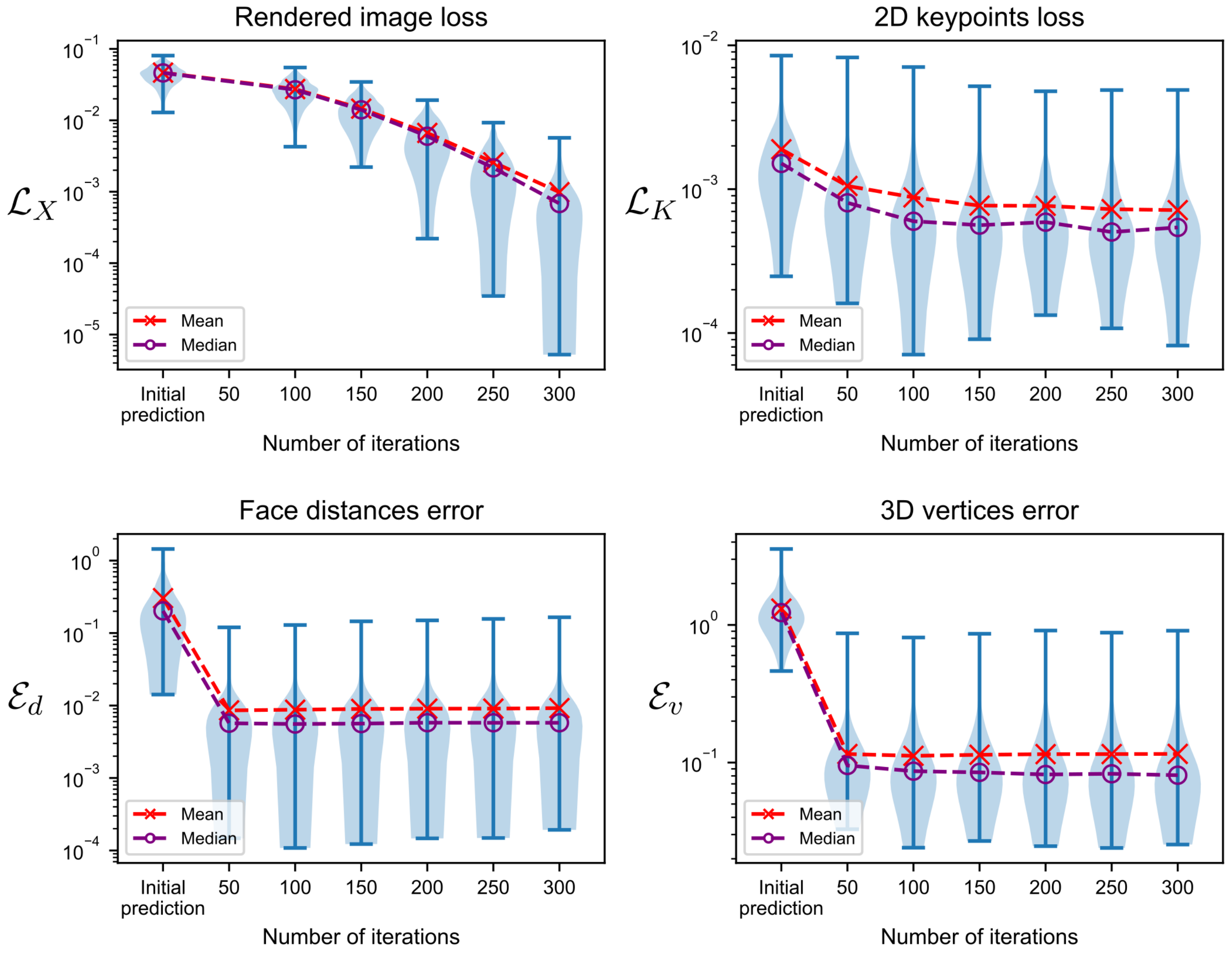

Fig. S3. Refinement iterations improve the initial predictions on synthetic data. To demonstrate this, we randomly selected 300 images from the withheld test portion of the synthetic dataset, made initial predictions and refined the parameters for 50 steps without inverse rendering and 250 steps with inverse rendering, using the method described in the main text. $\mathcal{L}_X$ and $\mathcal{L}_K$ are the image and keypoints loss terms respectively that form the principle minimisation targets. Here, since ground truth parameter values are known, we can also show the face distances errors $\mathcal{E}_d = \sum_i (d_i - d_i^*)^2/|D|$, where d_i^* is the target distance, and the errors between known and predicted 3D vertices; $\mathcal{E}_v = \text{mean}(\min_j \Lambda_{ij}, \min_i \Lambda_{ij})$ where $\Lambda_{ij} = \|v_i - v_j^*\|_2$ is the pairwise matrix of Euclidean distances from which we select just the nearest in both directions ($\min_j$ and $\min_i$). Face distance errors of $\sim 10^{-2}$ correspond roughly to the size of a single pixel. Although these are synthetic examples, the length units closely approximate millimetres due to the intentional design of the synthetic scene as a digital twin of the real-world setup.

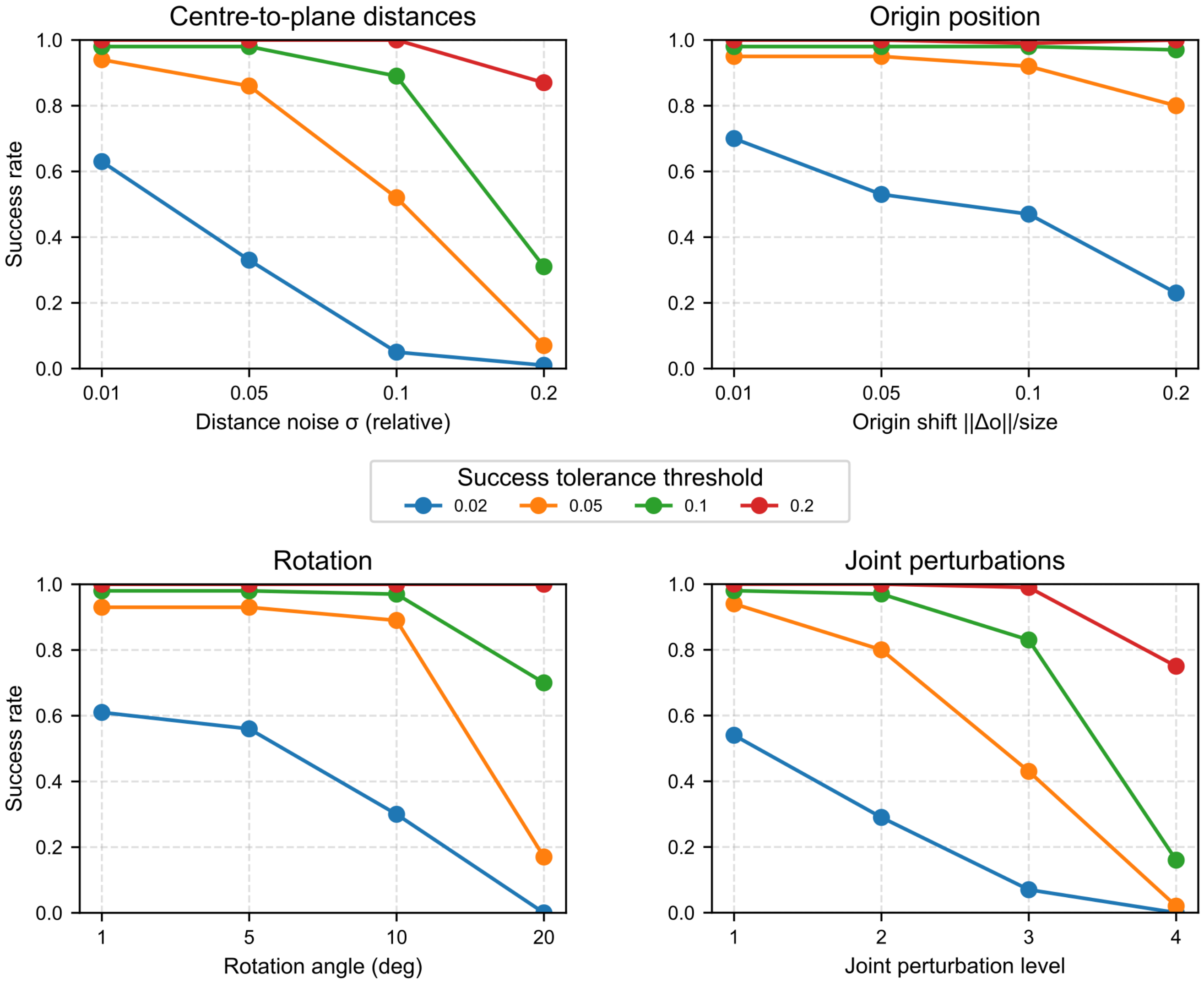

Fig. S4. Stability of inverse-rendering refinement to initialisation. For 100 randomly selected synthetic test images from the set previously refined in Fig. S3 (*i.e.* a subset of those 300 refined examples), we start a fresh refinement run using identical refinement settings (loss weights, optimisation hyperparameters, and the initial 50 steps without inverse-rendering) to the original synthetic refinement. Each run is initialised from the corresponding converged reference solution and then subjected to controlled random perturbations of: (i) centre-to-plane distances (multiplicative Gaussian noise with standard deviation $\sigma \in \{0.01, 0.05, 0.1, 0.2\}$), (ii) origin (random in-plane shift with $\|\Delta o\|/s \in \{0.01, 0.05, 0.1, 0.2\}$, where s is the largest centre-to-plane distance for each crystal), (iii) rotation (random-axis 3D rotation with angle $\Delta\theta \in \{1^\circ, 5^\circ, 10^\circ, 20^\circ\}$), and (iv) joint perturbations across all three parameter groups using matched levels. This yields 16 perturbation conditions per image (4 perturbation types × 5 levels), for 1600 refinement runs in total. Each subplot reports the fraction of runs that return to the reference solution under a tolerance $\tau \in \{0.02, 0.05, 0.1, 0.2\}$, where success is defined as a final normalised 3D vertex error $\mathcal{E}_v \leq \tau$, computed as the mean nearest-neighbour vertex distance normalised by s. Note that lack of "success" does not necessarily imply convergence to a different local optimum, only that the optimisation has not returned to the original solution after 300 steps. These results suggest that refinement is more robust to rigid-body misalignment than to perturbations of the face-distance (morphology) parameters, with compounded perturbations showing the expected lowest return rates.

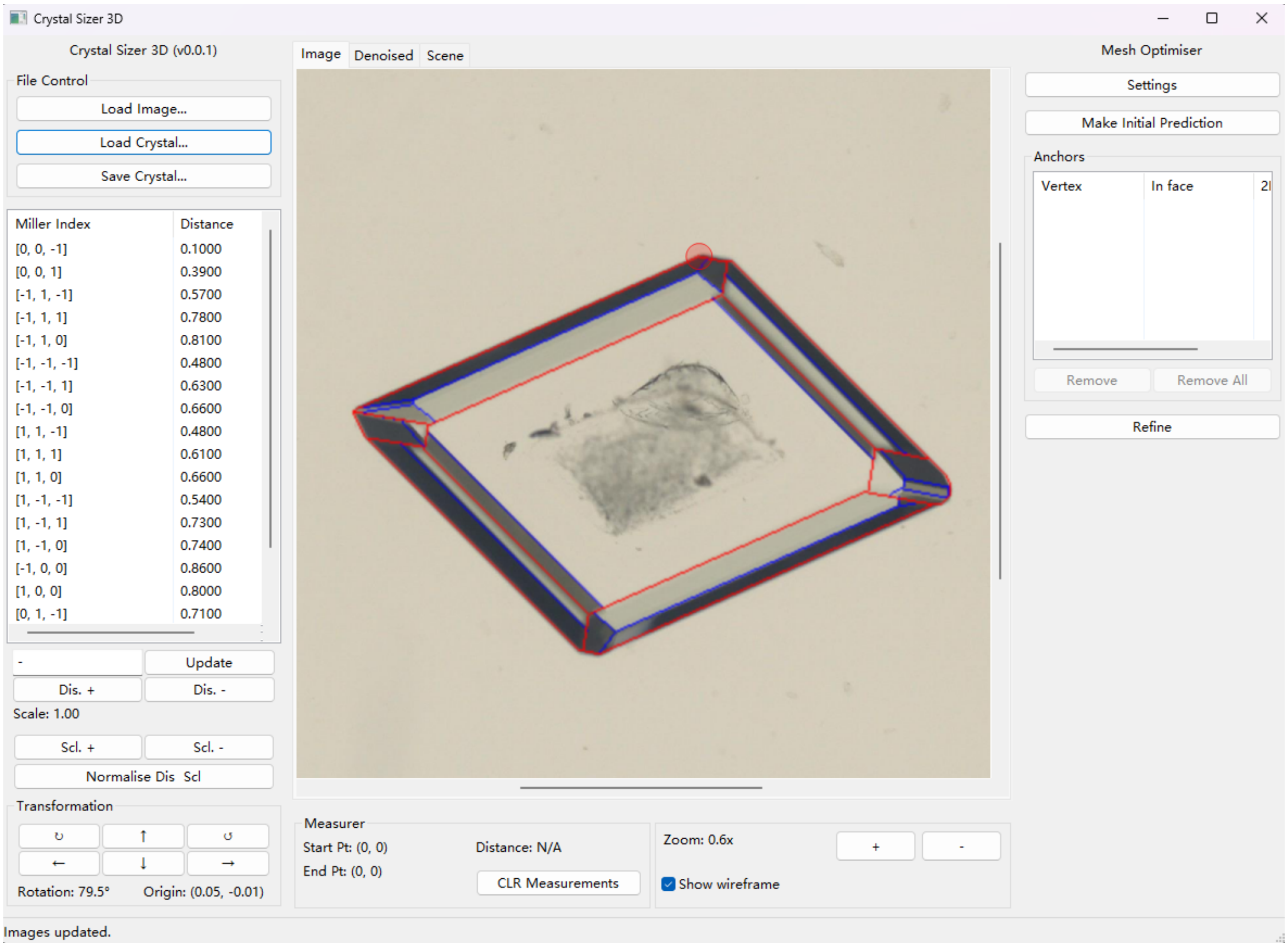

Fig. S5. A screenshot of the manual fitting user interface. Users can load images and crystal template files to visualise the matching of the projected, refracted wireframe against the real crystal. The configuration parameters (plane distances, origin position, rotation and refractive index) can be adjusted by hand to obtain a good visual fit.

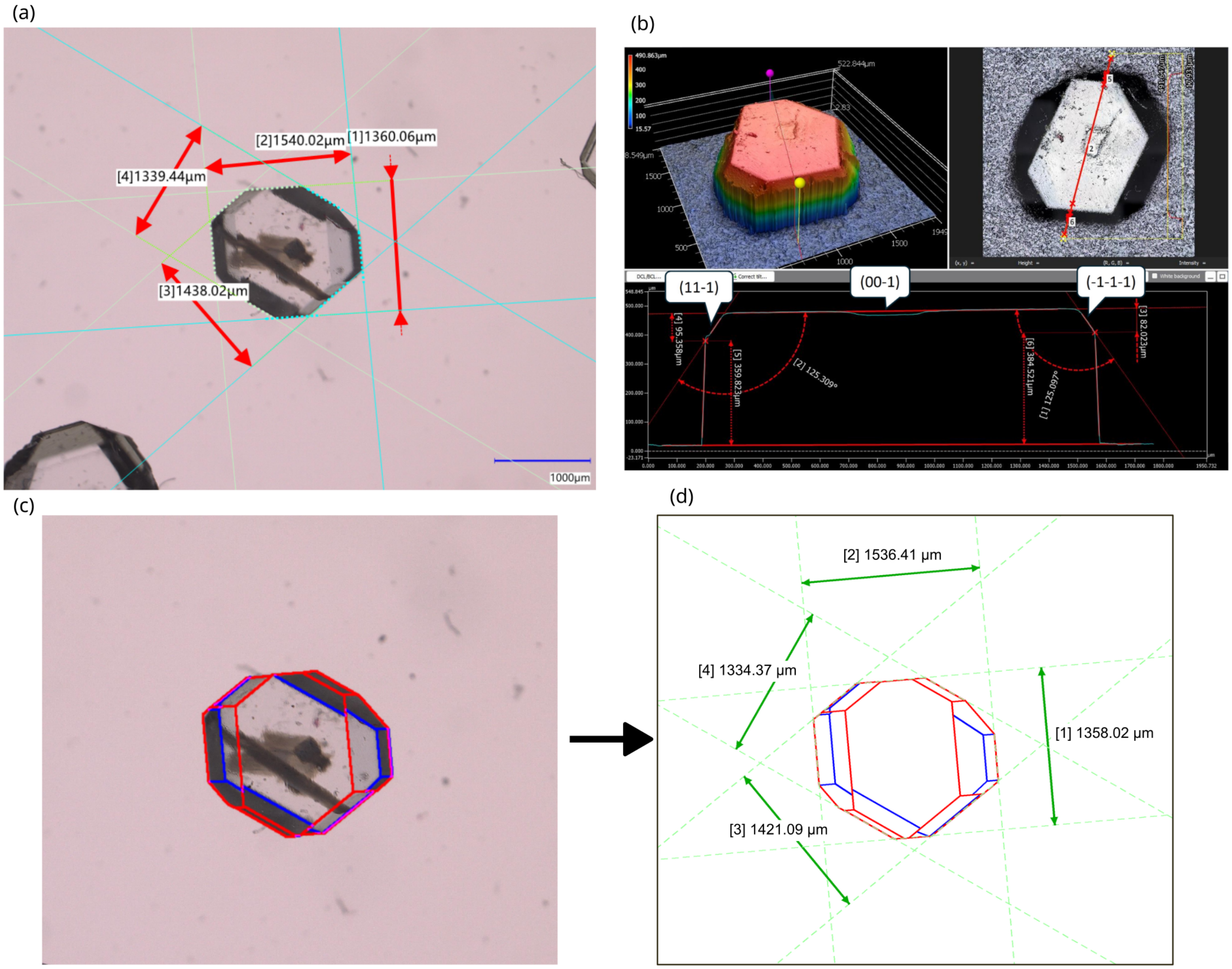

Fig. S6. Validation of the physical accuracy of the optical model and fitting software. (a) 2D crystal measurements calculated using the Keyence VHX7000 microscope software. The Keyence software tool helps users to align parallel lines to crystal edges and simplifies conversion of pixel distances to physical units, but provides no image processing, feature detection or automatic fitting. (b) Keyence VKX3000 confocal microscope measurements of the same crystal used to recover the crystal height. (c) Crystal mesh (with refraction) fitted using our software tool. (d) 2D measurements of the projected fitted crystal mesh can be easily calculated for comparison and validation with (a). Measurement comparisons are shown in Table S1. More details are included in Sec. S3.

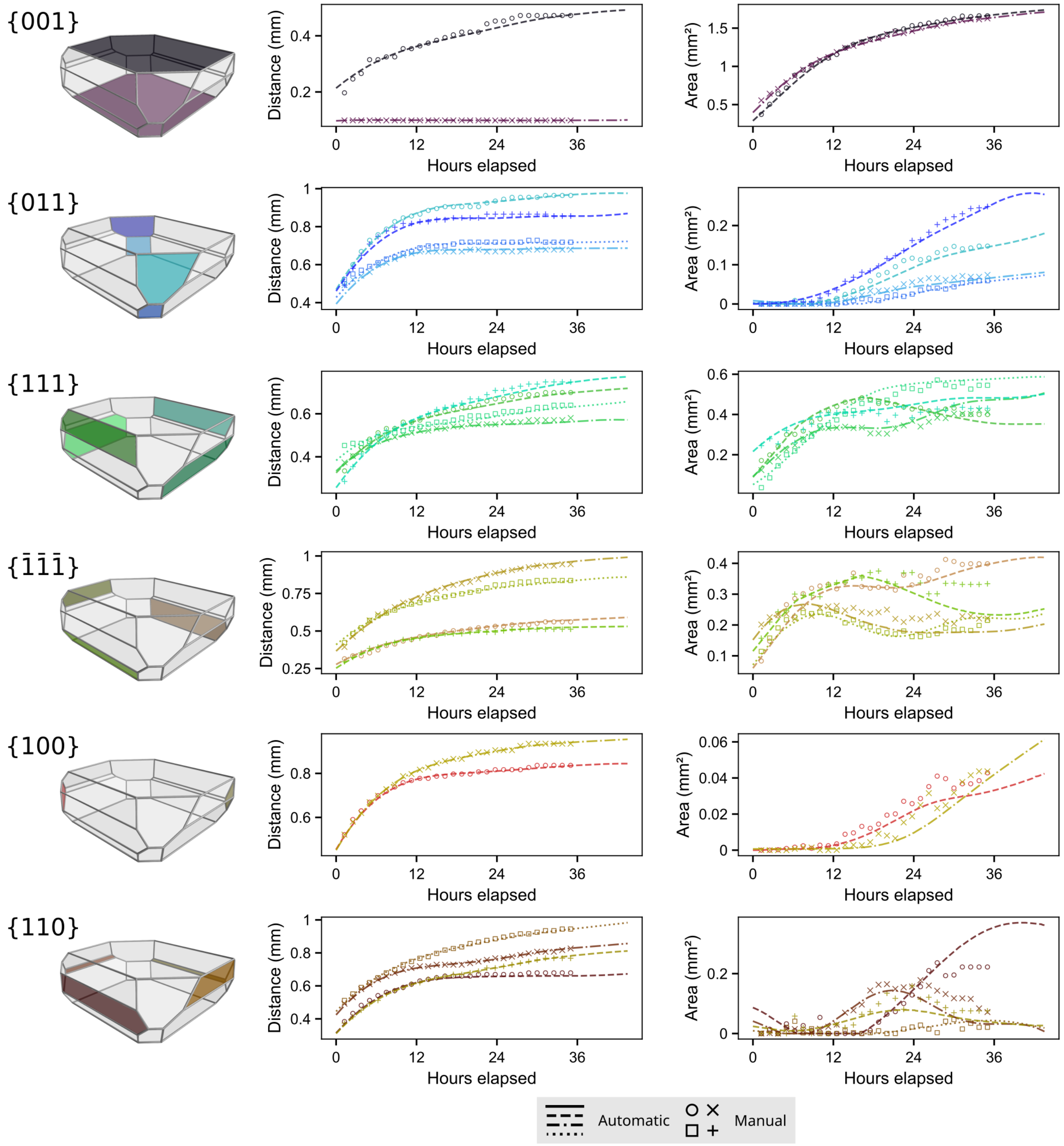

Fig. S7. Enlarged result plots from automatic fitting of a real crystal growth sequence (sequence 2 in Table S2), presented in Fig. 1 in the main text. Each row corresponds to a different face group. Origin-to-plane distances and face areas are displayed in the second and third columns, respectively. Lines represent the automatic measurements, symbols represent manual measurements.

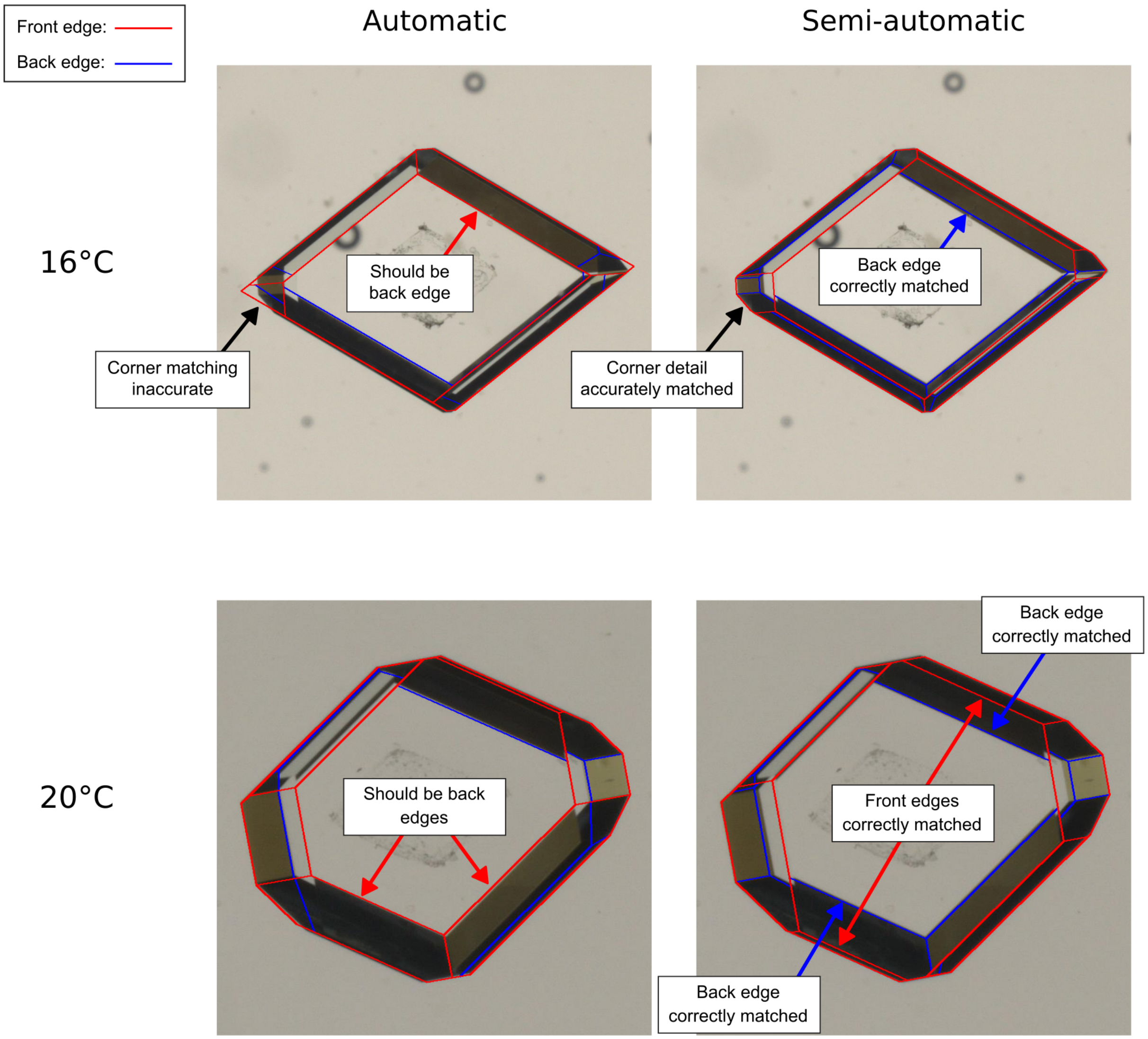

Fig. S8. The semi-automatic approach is able to improve upon partially-matched automatic fittings. The optimisation process for the semi-automatic results remains the same as for fully automatic, but with an additional loss term included that helps resolve ambiguities in the shape and thus recovers near-perfect matches (see Methods).

Table S1. Optical model validation

	Independent measurement (μm)	Our measurement A (μm)	Our measurement B (μm)	Difference (μm)	Relative difference (%)
2D in-plane measurements					
$(100) - (\bar{1}00)$ #1 in Fig. S6d	1360	1358	-	-2	-0.1
$(010) - (0\bar{1}0)$ #2 in Fig. S6d	1540	1536	-	-4	-0.2
$(110) - (\bar{1}\bar{1}0)$ #3 in Fig. S6d	1438	1421	-	-17	-1.2
$(1\bar{1}0) - (\bar{1}10)$ #4 in Fig. S6d	1339	1334	-	-5	-0.4
Axial measurements					
Total height	455 – 467	398	403	-68 – -53	-14.7 – -11.6
z-span $(\bar{1}\bar{1}\bar{1})$	82	136	140	54 – 58	39.9 – 41.4
z-span $(\bar{1}\bar{1}0)$	-	95	102	-	-
z-span $(\bar{1}\bar{1}1)$	140	167	160	20 – 27	14.4 – 19.2
z-span $(\bar{1}00)$	-	319	323	-	-
z-span $(\bar{1}1\bar{1})$	255	299	307	44 – 51	17.1 – 20.2
z-span $(\bar{1}10)$	-	30	-	-	-
z-span $(\bar{1}11)$	-	69	95	-	-
z-span $(0\bar{1}\bar{1})$	97 – 152	114	131	-38 – 34	-24.8 – 34.5
z-span $(0\bar{1}1)$	286	284	271	-14 – -2	-5.0 – -0.6
z-span $(01\bar{1})$	141 – 198	171	212	-27 – 71	-13.5 – 50.4
z-span (011)	231	227	190	-41 – -4	-17.9 – -1.7
z-span $(1\bar{1}\bar{1})$	318	307	333	-11 – 16	-3.5 – 4.9
z-span $(1\bar{1}1)$	-	91	69	-	-
z-span (100)	-	398	398	-	-
z-span $(11\bar{1})$	95	154	141	45 – 59	47.5 – 61.9
z-span (110)	-	73	115	-	-
z-span (111)	165	170	146	-19 – 5	-1.1 – 3.1

In addition to the total crystal height, we report multiple out-of-plane z-span "independent measurement" values corresponding to distinct height intervals between the top face and the termination of the sloping faces visible in the laser confocal cross-section (and repeated with a flipped crystal to recover the rear sloping faces). Confocal heights are reported as ranges where the software readout varies across the sampled cross-section or differs across multiple measurements of the same heights. "Our measurement" values are computed from the 3D mesh fitted using our software tool (Fig. S5): 2D values are obtained by projecting the fitted mesh into the image plane and extracting the corresponding edge-to-edge distances (panel (d) in Fig. S6), and z-span values are obtained by computing the corresponding out-of-plane z-extents of the relevant faces from the 3D mesh. Two manual fits were performed independently on images of the original crystal (A) and the flipped crystal (B). Yellow cells indicate front faces, blue cells indicate rear faces. Difference values are "worst-case" when comparing ranges. More details included in Sec. S3.

Table S2. Mean absolute errors on nine crystal growth sequences

**Growth sequence**	**Temperature** °C	**# manually fitted images /total # images**	**Volume** MAE (std) mm^3	**Surface area** MAE (std) mm^2	**Face area** MAE (std) mm^2	**Plane distance** MAE (std) mm
1	12	6/200	0.034 (0.017)	0.115 (0.059)	0.017 (0.023)	0.011 (0.010)
2	14	28/522	0.025 (0.016)	0.104 (0.065)	0.023 (0.030)	0.008 (0.008)
3	16	6/200	0.852 (0.360)	3.037 (0.920)	0.271 (0.027)	0.124 (0.149)
			0.022 (0.013)	*0.097 (0.039)*	*0.011 (0.014)*	*0.008 (0.007)*
4	18	6/200	0.045 (0.031)	0.137 (0.085)	0.021 (0.024)	0.013 (0.014)
5	20	8/200	0.016 (0.013)	0.056 (0.057)	0.023 (0.027)	0.012 (0.010)
6	20	8/200	0.041 (0.026)	0.211 (0.141)	0.032 (0.039)	0.027 (0.036)
			0.012 (0.009)	*0.084 (0.058)*	*0.006 (0.010)*	*0.007 (0.006)*
7	20	6/200	0.091 (0.025)	0.356 (0.129)	0.030 (0.024)	0.013 (0.017)
8	22	6/200	0.077 (0.067)	0.274 (0.187)	0.021 (0.019)	0.014 (0.014)
9	26	7/200	0.022 (0.013)	0.074 (0.031)	0.029 (0.037)	0.011 (0.010)

Mean absolute errors (with standard deviations in brackets) across the nine experiments relative to the manually-fitted crystals. Sequences 3 and 6 were only partially-correct fits, with some front/back edges confused (see Fig. S8). Semi-automatic fitting results for sequences 3 and 6 are shown beneath, in italics.

Table S3. Synthetic crystal dataset parameters

Parameter	Value/Range
Crystal	
Crystal ID (in the Crystal Structures Database, CSD (5))	LGLUAC02
Symmetric distance ($\bar{d}$) constraints	$\begin{cases} \bar{d}_{011} > \bar{d}_{001} > 0 \\ \bar{d}_{111} > \bar{d}_{001} > 0 \\ \bar{d}_{\bar{1}\bar{1}\bar{1}} > \bar{d}_{001} > 0 \\ \bar{d}_{100} > \bar{d}_{001} > 0 \\ \bar{d}_{110} > \bar{d}_{001} > 0 \end{cases}$
Symmetric distance ($\bar{d}$) scale-invariant means	$\begin{cases} \mu_{001} = 1 \\ \mu_{011} = 1 \\ \mu_{111} = 1 \\ \mu_{\bar{1}\bar{1}\bar{1}} = 1 \\ \mu_{100} = 1 \\ \mu_{110} = 1.5 \end{cases}$
Symmetric distance ($\bar{d}$) scale-invariant standard deviations	$\begin{cases} \sigma_{001} = 1 \\ \sigma_{011} = 1 \\ \sigma_{111} = 1 \\ \sigma_{\bar{1}\bar{1}\bar{1}} = 1 \\ \sigma_{100} = 1 \\ \sigma_{110} = 1 \end{cases}$
Distance asymmetry (relative average)	0.1
Zingg shape characterisation bounds ((15)) on the minimal 3D bounding cuboid with dimensions $a \leq b \leq c$	$\begin{cases} 0.2 < \frac{c}{b} < 1 \\ 0.2 < \frac{b}{a} < 1 \end{cases}$
Maximum rotation out of xy plane ($\max(R_x)$, $\max(R_y)$)	0.05
Index of refraction, η	$[1.3, 1.9]$
Roughness, r	$[0.03, 0.25]$
Image area (relative), a_{target}	$[0.012, 0.3]$
Surface defects	
Number	$[0, 10]$
Width	$[1e^{-4}, 2e^{-3}]$
Internal seed crystal	
Probability of inclusion	0.8
Scale-invariant distances variance	0.1
Scale (relative to main crystal)	$[0.1, 0.6]$
Origin variance (from centre of main crystal)	0.1
Texture dimension	1000×1000 pixels
Texture amplitude	$[0.01, 0.4]$
Perlin frequency	$[3, 8]$
Perlin octaves	$[4, 10]$
White noise scale	$[1e^{-4}, 5e^{-3}]$
Bubbles	
Number	$[0, 20]$
Refractive index, η	$[1.3, 2.3]$
Roughness, r	$[0.05, 0.2]$
Scale, s	$[0.001, 0.1]$
Cell surface	
Bumpmap dimension	1000×1000 pixels

Parameter	Value/Range
Noise amplitude	$[0.01, 0.5]$
Perlin frequency	$[0.1, 10]$
Perlin octaves	$[1, 10]$
White noise scale	$[0.01, 0.2]$
Light	
Radiance, l	$[0.4, 0.8] \times 3$
Radiance maximum range, $\max_{ij}\{l_i - l_j\}$	0.1
Texture dimension	1000×1000 pixels
Noise amplitude	$[0.01, 0.3]$
Perlin frequency	$[0.1, 4]$
Perlin octaves	$[1, 10]$
White noise scale	$[0.01, 0.2]$
Rendering settings	
Dataset size	$100,000$
Image size	400×400 pixels
Samples per pixel	324
Sampler type	stratified
Integrator maximum depth	32

Table S4. Neural network training parameters

Parameter	Predictor	Denoiser	Keypoint Detector
	Image augmentation		
Probability of adding a Gaussian blur	0.3	0.3	0.3
Gaussian blur sigma ($\sim$ amount)	$[0.01, 5.0]$	$[0.01, 5.0]$	$[0.01, 5.0]$
Probability of adding Gaussian-distributed noise	0.3	0.3	0.3
Gaussian noise sigma ($\sim$ amount)	$[0.01, 0.1]$	$[0.01, 0.1]$	$[0.01, 0.1]$
	Optimiser		
Optimiser	AdamW (8)	AdamW	AdamW
Initial learning rate	$1e^{-4}$	$1e^{-5}$	$1e^{-5}$
Final learning rate	$1e^{-5}$	$1e^{-6}$	$1e^{-6}$
Learning rate decay	cosine	cosine	cosine
Weight decay	$1e^{-3}$	$1e^{-3}$	$1e^{-3}$
Clip gradient norm	1	1	1
Batch size ($\times$ gradient accumulation)	32	4×2	4×2
	Loss weightings		
ω_{v}	1.0		
ω_{d}	1.0		
ω_{m}	0.1		
ω_{l}	0.033		

Table S5. Predictor network architecture evaluation

**Model**	**# Parameters**	$\mathcal{L}_\mathsf{d}$	$\mathcal{L}_\mathsf{p}$	$\mathcal{L}_\mathsf{m}$	$\mathcal{L}_\mathsf{l}$	$(\mathcal{L}_\mathsf{v})$	$\mathcal{E}_d$ (mm)	$\mathcal{E}_v$ (mm)
RegNetZ (11)	21.70	**0.0035**	**0.0598**	**0.1820**	0.0394	**0.0611**	0.0882	0.1920
RegNetX (11)	6.63	0.0048	0.0790	0.3015	0.0667	0.0742	0.1659	0.3298
RegNetY (11)	80.76	**0.0035**	0.0642	0.2440	**0.0318**	0.0614	**0.0659**	0.1817
EfficientNet-B3 (13)	10.79	0.0042	0.0691	0.2460	0.0517	0.0671	0.1884	0.3774
EfficientNet-B4 (13)	17.66	0.0047	0.0746	0.3017	0.0645	0.0732	0.1960	0.3168
ECA-ResNet-D (14)	100.18	0.0036	0.0639	0.1949	0.0410	0.0629	0.0699	**0.1803**
MobileNet-V4-Small (10)	2.58	0.0051	0.0862	0.3963	0.0796	0.0800	0.3079	0.4793
MobileNet-V4-Large (10)	31.39	0.0105	0.0912	0.4222	0.3248	0.1053	0.1209	0.2745
ViT-Small (4)	21.86	0.0053	0.0890	0.3692	0.0362	0.0748	0.0942	0.2698
ViT-Base (4)	86.18	0.0055	0.0947	0.4431	0.0320	0.0751	0.1659	0.3328
ViT-RelPos (7)	118.58	0.0060	0.1081	0.4944	0.0443	0.0766	0.0844	0.6985

One-shot prediction performance, best values in bold. Models were trained on the synthetic crystal images dataset for up to 100 epochs or 48 hours, whichever came first. Reported are the losses on the withheld test set (20,000 examples) and subsequent performance on 28 manually measured real crystal images. Test losses: centre-to-plane distances ($\mathcal{L}_\mathsf{d}$), pose ($\mathcal{L}_\mathsf{p} = \mathcal{L}_\mathsf{P} + \mathcal{L}_\mathsf{R}$), materials ($\mathcal{L}_\mathsf{m}$), light ($\mathcal{L}_\mathsf{l}$) and 3D vertex positions ($\mathcal{L}_\mathsf{v}$). Note that $\mathcal{L}_\mathsf{v}$ is not used for training. Measurement errors: centre-to-plane distances ($\mathcal{E}_d$) and 3D vertex positions ($\mathcal{E}_v$). See main text for more details.

Table S6. Refinement parameters

Parameter	Value/Range
Denoising settings	
Number of tiles	9
Tile overlap	8 %
Model input size (tiles are downsampled to this)	512×512
Keypoint detection settings	
Number of patches	9
Patch size	700×700
Exclude border	5 %
Model input size (patches are downsampled to this)	512×512
Minimum distance between keypoints (merge threshold at full resolution)	20 px
Rendering settings	
Rendering size	400×400
Samples per pixel	32
Integrator max. depth	8
Pretraining	
Optimiser	AdamW (8)
Learning rate	$1e^{-3}$
Weight decay	$1e^{-3}$
Clip gradient norm	10
Batch size	256
Steps	1000
Sequence fitting	
Optimiser	AdamW (8)
Initial learning rate	$1e^{-4}$
Minimum learning rate	$1e^{-5}$
Learning rate decay	plateau
Learning rate decay rate	0.8
Learning rate decay patience	200 steps
Weight decay	$1e^{-4}$
Clip gradient norm	10
Batch size	32
Loss weightings	
ω_X	1
ω_K	10
ω_z	1
ω_R	1
ω_o	10
ω_+	100

Table S7. Processing timings and hardware requirements

**Component**	**Laptop time** seconds (std)	**HPC time** seconds (std)	**Laptop peak GPU memory** GiB	**HPC peak GPU memory** GiB
		One-shot / Components		
Denoiser	0.880 (0.009)	0.410 (0.001)	2.93	2.93
Keypoint detector	2.106 (0.016)	1.036 (0.001)	3.95	3.95
Predictor	0.017 (0.006)	0.010 (0.003)	0.31	0.31
One-shot total	3.003	1.456	3.95	3.95
		Single-frame refinement – 100 steps		
Keypoints-only	15.03 (0.48)	15.73 (0.16)	2.52	2.52
Full	108.00 (5.99)	119.49 (5.52)	4.65	4.65
		Sequence fitting – hours		
Pretrain (1000 steps)	–	0.01	–	–
Keypoints-only (1000 steps)	–	0.41	–	–
Full (2000 steps)	–	3.88	–	–
Total (3000 steps)	–	4.32	–	–

Runtime and GPU memory usage for the main pipeline components on a laptop GPU (NVIDIA GeForce RTX 4090; 16 GB VRAM) and an HPC GPU node (NVIDIA L40S; 48 GB VRAM). Times are reported as mean (standard deviation) over 20 frames for per-image/per-frame benchmarks using the parameter values listed in Table S6. Component timings measure GPU computation with images preloaded in memory (excluding disk I/O); refinement timings include both initialisation and optimisation. Sequence fitting timings are derived from HPC run logs; we therefore report them only under the HPC column. Reported memory is peak GPU memory allocated during the timed region and is therefore indicative; actual runtimes and memory use depend on hardware and image resolution, and can vary significantly with different optimisation settings (iterations/steps, rendering parameters, *etc.*). These measurements are provided to give a practical order-of-magnitude estimate; the open-source implementation prioritises research flexibility over performance tuning.

Movie S1. Denoising and keypoint detection

A crystal growth sequence is displayed across three panels that display, from left to right: a) The original microscope images. b) The denoised image sequence. c) The detected keypoints (green circles) overlaid onto the original images.

Movie S2. Initial predictions

A crystal growth sequence is displayed across three panels that display, from left to right: a) The original microscope images. b,c) The results of the one-shot prediction of the 3D crystal shapes. In (b) the predicted 3D wireframes are projected (with refraction) onto the original images. In (c) the predicted 3D shapes are rendered into new synthetic images.

Movie S3. Sequence fitting (refinement)

A crystal growth sequence is displayed across three panels that display, from left to right: a) The original microscope images. b,c) The results of the sequence fitter after refining the initial predictions using keypoint and inverse rendering optimisation. In (b) wireframes of the refined 3D shapes are projected (with refraction) onto the original images. In (c) the refined 3D shapes are rendered into new synthetic images.

Movie S4. Combined results

A crystal growth sequence is displayed across six panels that display in two rows. The top row displays, from left to right: a) The original microscope images. b,c) Wireframes of the initial (b) or refined (c) 3D shapes projected (with refraction) on to the original images. The bottom row displays, from left to right: d) The detected keypoints (green circles) overlaid onto the original images. e,f) Synthetic renderings of the initial (e) or refined (f) 3D shapes.

Movie S5. Semi-automatic results

Two crystal growth sequences are displayed across six panels that display in two rows. The rows correspond to the experiments shown in Fig. S8 and demonstrate how partially-correct automatic fits can be nudged to the correct solution by including a small number of manual measurements into the optimisation. The rows display, from left to right: a) The original microscope images. b) Wireframes of the automatic fit 3D shapes projected on to the original images. c) Wireframes of the semi-automatic fit 3D shapes projected on to the original images.

SI Dataset S1. Synthetic α-LGA crystal dataset

The synthetic dataset that was used for training the denoiser, keypoint detector and initial predictor networks, along with the experimental data and the videos described above is available here: https://doi.org/10.5518/1684. Below is an overview of the dataset contents:

- **crystal_bumpmaps/** – Normal-map-style randomised bumpmaps for each crystal to be applied the surface of the crystal objects as a texture.
- **ds_stats.yml** – Summary statistics for the dataset, including minimums, maximums, means and variances for all of the parameters.
- **experiments/** – Original microscope images and manual measurements for the nine experiments reported in the paper.
- **images/** – RGB renderings of synthetic crystal configurations including randomised sources of noise.
- **images_clean/** – RGB renderings of synthetic crystal configurations without randomised sources of noise.
- **keypoints_wfv=1.0_kpv=1.0/** – Generated 3D keypoint and wireframe heatmaps stored as three-channel images where the first channel contains Gaussian blobs of variance 1 px at each keypoint location, the second channel contains all of the camera-facing wireframe edges and the third contains all of the refracted wireframe edges.
- **keypoints_wfv=1.0_kpv=10.0/** – As above, but with larger variance (10 px) used to generate the Gaussian blobs at the keypoint locations.
- **options.yml** – Full configuration settings used for dataset generation and synthetic image rendering (values also listed in Table S3).
- **parameters.csv** – Crystal configuration parameters, canonical distances, scale, image references and computed face areas.
- **rendering_parameters.json** – Detailed representation of each synthetic crystal scene including randomised lighting configurations, randomised textures and bumpmap generation parameters and crystal seed configurations.
- **train_test_split_0.80.json** – Dataset sample indices for training and testing split (80/20), used in all experiments.
- **validation/** – Additional validation images included to verify that the dataset images can be completely reconstructed from the data saved in 'rendering_parameters.json'.

- **vertices.json** – 3D mesh vertex data for each synthetic crystal, used as ground truth for geometric losses.
- **videos/** – Videos files demonstrating the method and results as described above.

References

1. Max Born and Emil Wolf. *Principles of Optics: Electromagnetic Theory of Propagation, Interference and Diffraction of Light*. Cambridge University Press, 7th edition, 2020. 60th Anniversary Edition.
2. Stephen Dawson-Haggerty et al. trimesh: A python library for loading and using triangular meshes. https://trimesh.org/.
3. Bram de Greve. Reflections and refractions in ray tracing, 2006. https://graphics.stanford.edu/courses/cs148-10-summer/docs/2006--degreve--reflection_refraction.pdf [Accessed: (7 April 2025)].
4. Alexey Dosovitskiy, Lucas Beyer, Alexander Kolesnikov, Dirk Weissenborn, Xiaohua Zhai, Thomas Unterthiner, Mostafa Dehghani, Matthias Minderer, Georg Heigold, Sylvain Gelly, et al. An image is worth 16x16 words: Transformers for image recognition at scale. *arXiv preprint arXiv:2010.11929*, 2020.
5. Colin R Groom, Ian J Bruno, Matthew P Lightfoot, and Suzanna C Ward. The cambridge structural database. *Structural Science*, 72(2):171–179, 2016.
6. Maya Khellaf, Catherine Charcosset, Denis Mangin, and Elodie Chabanon. Solubility of l-glutamic acid in concentrated water/ethanol solutions. *Journal of Crystal Growth*, 570:126238, 2021.
7. Ze Liu, Han Hu, Yutong Lin, Zhuliang Yao, Zhenda Xie, Yixuan Wei, Jia Ning, Yue Cao, Zheng Zhang, Li Dong, et al. Swin transformer v2: Scaling up capacity and resolution. In *Proceedings of the IEEE/CVF conference on computer vision and pattern recognition*, pages 12009–12019, 2022.
8. Ilya Loshchilov and Frank Hutter. Decoupled weight decay regularization. *arXiv preprint arXiv:1711.05101*, 2017.
9. Cai Y Ma, Chen Jiang, Thomas P Ilett, Thomas A Hazlehurst, David C Hogg, and Kevin J Roberts. Deconstructing 3d growth rates from transmission microscopy images of facetted crystals as captured in situ within supersaturated aqueous solutions. *Journal of Applied Crystallography*, 57(5):1557–1565, 2024.
10. Danfeng Qin, Chas Leichner, Manolis Delakis, Marco Fornoni, Shixin Luo, Fan Yang, Weijun Wang, Colby Banbury, Chengxi Ye, Berkin Akin, et al. Mobilenetv4: universal models for the mobile ecosystem. In *European Conference on Computer Vision*, pages 78–96. Springer, 2024.
11. Ilija Radosavovic, Raj Prateek Kosaraju, Ross Girshick, Kaiming He, and Piotr Dollár. Designing network design spaces. In *Proceedings of the IEEE/CVF conference on computer vision and pattern recognition*, pages 10428–10436, 2020.
12. Michael T Ruggiero, Juraj Sibik, J Axel Zeitler, and Timothy M Korter. Examination of l-glutamic acid polymorphs by solid-state density functional theory and terahertz spectroscopy. *The Journal of Physical Chemistry A*, 120(38):7490–7495, 2016.
13. Mingxing Tan and Quoc Le. Efficientnet: Rethinking model scaling for convolutional neural networks. In *International conference on machine learning*, pages 6105–6114. PMLR, 2019.
14. Qilong Wang, Banggu Wu, Pengfei Zhu, Peihua Li, Wangmeng Zuo, and Qinghua Hu. Eca-net: Efficient channel attention for deep convolutional neural networks. In *Proceedings of the IEEE/CVF conference on computer vision and pattern recognition*, pages 11534–11542, 2020.
15. Theodor Zingg. *Beitrag zur schotteranalyse*. PhD thesis, ETH Zurich, 1935.
